# Misinformation vs. Situational Awareness: The Art of Deception and the Need for Cross-Domain Detection

**DOI:** 10.3390/s21165496

**Published:** 2021-08-15

**Authors:** Constantinos-Giovanni Xarhoulacos, Argiro Anagnostopoulou, George Stergiopoulos, Dimitris Gritzalis

**Affiliations:** Department of Informatics, Athens University of Economics & Business, 10434 Athens, Greece; cxarhou@aueb.gr (C.-G.X.); anagnostopouloua@aueb.gr (A.A.); geostergiop@aueb.gr (G.S.)

**Keywords:** situational awareness, cyber situational awareness, misinformation, cybersecurity, fake news, Information and Communication Technology (ICT) security, incident response, deception detection, Cross-Domain detection

## Abstract

The world has been afflicted by the rise of misinformation. The sheer volume of news produced daily necessitates the development of automated methods for separating fact from fiction. To tackle this issue, the computer science community has produced a plethora of approaches, documented in a number of surveys. However, these surveys primarily rely on one-dimensional solutions, i.e., deception detection approaches that focus on a specific aspect of misinformation, such as a particular topic, language, or source. Misinformation is considered a major obstacle for situational awareness, including cyber, both from a company and a societal point of view. This paper explores the evolving field of misinformation detection and analytics on information published in news articles, with an emphasis on methodologies that handle multiple dimensions of the fake news detection conundrum. We analyze and compare existing research on cross-dimensional methodologies. Our evaluation process is based on a set of criteria, including a predefined set of performance metrics, data pre-processing features, and domains of implementation. Furthermore, we assess the adaptability of each methodology in detecting misinformation in real-world news and thoroughly analyze our findings. Specifically, survey insights demonstrate that when a detection approach focuses on several dimensions (e.g., languages and topics, languages and sources, etc.), its performance improves, and it becomes more flexible in detecting false information across different contexts. Finally, we propose a set of research directions that could aid in furthering the development of more advanced and accurate models in this field.

## 1. Introduction

Social networks and online news reading have undoubtedly become a staple of our daily routine. However, the ease of access to any type of information provides fertile ground for the systematic spread of falsehoods through “informative” websites or even trusted news outlets. The term “news” includes information in the form of articles, claims, statements, speeches, or posts that can be generated by any individual (journalist or otherwise). The term “fake news” refers to intentionally false news that has been published by a news source [1]. 

Fake news is a current problem that affects the world negatively. It is a multi-faceted phenomenon that can be split into different categories based on its contents and the purpose it serves. Fake news has appeared under several definitions, depending on the individual’s point of view. Gelfert defines fake news as “purportedly factual claims that are epistemically deficient (in a way that needs to be specified)” [2]. Tandoc E. et al. refer to fake news as “news articles that are intentionally and verifiably false and could mislead readers” [3]. Either definition could be considered correct depending on perspective; however, the consensus regarding the matter is quite clear. Information described by this term is considered partially or completely fictitious, often sensationalistic and backed by minimal corroboratory evidence. Current trends in misinformation show a growing amount of false news being spread and a growing need for society to increase its situational awareness in order to be able to distinguish real news from misinformation. 

Situational Awareness (SA) refers to knowledge that is publicly available and can be used to form an opinion so as to cope with a situation [4]. Existing literature contains numerous concepts that constitute the groundwork for the generation of misinformation. For instance, deceptive news, false/fake news, satirical news, disinformation, misinformation, cherry-picking, clickbait, and rumors are some of the most common [1]. We can differentiate these concepts based on: (1) authenticity, (2) intention, and (3) whether the information is news. For instance, false news contains non-factual statements, and its intention is undefined, while its information refers to actual news [1].

The computer science community has conducted research and devised methods able to detect false information successfully and efficiently. Among other technologies, machine learning has been effective for creating such automated frameworks. Specifically, the main approaches include [5]: 

Text analysis, including natural language processing (NLP)-based data representation, psychological feature analysis, syntax-based methods, and non-linguistic methods:

(1) Reputation/Publishing history analysis;
(2) Network data analysis;
(3) Image-based analysis;
(4) Image tampering detection.

Current issues in this research area include factors such as the linguistic and semantic features of misinformation (i.e., related to the writing style), scarce datasets, or the lack of a consistent data formatting, which increase the difficulty of detecting deception and misinformation. Existing models are characterized by poor adaptability in Cross-Domain tasks because the majority of them are often trained and tested on one specific source or domain of knowledge. This type of training makes the classifiers sensitive to noise [6]. Thus, there is room for improvement in the research, so as to develop more flexible methodologies. Cross-dimensional methods use different media sources, and thus, different content generators, along with diverse topics and fields [6]. Hence, these techniques and methodologies will be able to effectively detect fake news under different dimensions of data. 

### 1.1. Motivation 

This study focuses on the Cross-* methodologies for the deception detection of misinformation and the resulting situational awareness support. Cross-* includes all the categories of methodologies and frameworks that are not limited to one facet. All presented approaches fall into one of the following groups: (1) Cross-Domain Methodologies, (2) Cross-Language Methodologies, (3) Cross-Source Methodologies, and (4) Multi-Cross Methodologies. The first category refers to methods that can detect misinformation in more than one domain. The second category includes the methods that use datasets with two or more languages. The third category consists of methods that use data from more than one source, while the fourth one includes all those methods that combine two or more of the previous categories. 

Currently, the field of Cross-Domain, Cross-Language and Cross-Source misinformation detection is lacking when compared to traditional single-dimensional deception detection. Most publications focus on a singular aspect of the issue. Shifting the focus to cross-dimensional research would allow for a more thorough and extensive study of the problem and provide more comprehensive results on the matter [1]. Our goal is to map out the field of cross-* solutions for the rising problem of deception detection. We attempt to uncover and present those methods that cover more than one aspect of the problem. Afterwards, we conduct a thorough comparison of these solutions. We present a clear view of the status quo, in order to provide a benchmark for future work in this evolving field.

### 1.2. Contribution 

In this paper, we provide a thorough comparison of methodologies that are not limited to one dimension when opting to detect misinformation. Specifically, we conduct a thoughtful literature review to discover cross-dimensional deception detection methodologies for fake news. The term “cross-dimensional” refers to methods and frameworks that combine different sources, domains, or even different languages in the phases of training and evaluation of a model. 

Moreover, we assess the adaptability of each approach. Adaptability defines the degree to which a method can detect misinformation from different domains, languages, or sources. We also compare the four above mentioned cross-categories as a whole. 

Finally, we introduce good practices—research directions to establish a benchmark that may assist in furthering research in this nascent field and encourage the development of more accurate models.

### 1.3. Structure

The paper is structured as follows: Section 2 presents our research methodology along with a few related surveys associated with fake news detection. Section 3 provides the building blocks and groundwork for the machine learning techniques used in the detection of misinformation, along with metrics that are necessary to evaluate a machine learning technique. 

We also explain the two more complex criteria that we utilized during the comparison of the cross-* methodologies. Section 4 introduces a list of well-known datasets, along with the cross-* methods that are used in fake news detection among different domains, languages, and sources. The methods of each cross-category are also compared. 

Moreover, we present additional cross-* datasets as potential material for future work. In Section 5, a comparison of the four cross-categories is presented. Finally, in Section 6, we discuss the gaps that we identified in the existing approaches and propose a set of research directions. We also state our conclusions pointing out the limitations of deception detection.

## 2. Literature Review

Our survey approach includes four basic steps: (1) develop the research protocol, (2) discover the studies based on scope, (3) go through a screening of the literature regarding the quality criteria that are defined in the research protocol, and (4) extract and analyze information that are found to be in scope. These four steps offer a reproducible algorithm for handling scientific literature used in this survey for discovering cross-dimensional deception detection methodologies for fake news. 

**Figure 1:**
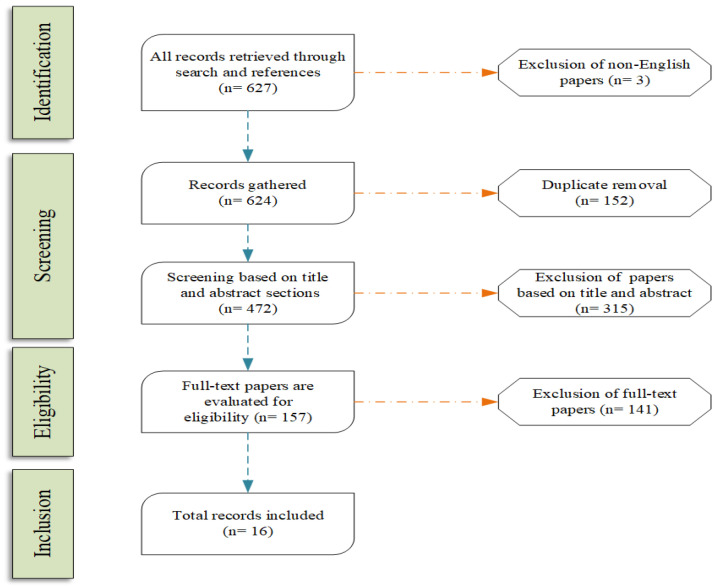
Survey method structure.

Figure 1 presents the flow of the steps that describe our survey method, which is based on PRISMA [7]. The first step was to gather all the documents (627 files) that has been detected in the academic field. We excluded articles written in languages we could not parse, removed duplicates, and moved on the evaluation of each detection. Some articles were excluded based on their title and their abstract (exclusion of 315 files), while other were rejected upon reading the full text body (exclusion of 141 files). The most common issue we faced was the detection of information that was tightly coupled with cross-dimensional fake news methods, and not a generic deception detection framework that applied to only one domain, language, or source. Finally, we included 16 articles in the core part of the survey. It is worth mentioning that we used additional literature for peripheral information of our survey, such as information about the presented datasets. We did not take them into account to the number of included files since they were not directly associated with our research questions.

We first define the scope of the survey. Then, we analyze and compare the available cross-* methodologies relevant with the detection of fake news. Table 1 presents the objectives along with their supporting research questions. Specifically, for each search goal, the relevant question posed to achieve this goal, as well as the related keyword searches used to detect relevant material, are presented. Our systematic literature search was conducted from January to April 2021. 

Preliminary findings were subsequently recorded in January 2021. The search engines utilized were Scopus, IEEE Xplore, and Google Scholar. Searches were carried out using a wide range of keywords and their combinations and were subjected to filtering and fine-tuning based on the context of results. Additional citations were also extracted from the google scholar algorithm that proposes relevant bibliography for each search [8].

### 2.1. Publications 

The search queries yielded a significant number of publications and literature. Hence, we defined a set of inclusion and exclusion criteria to evaluate the validity of the content and reduce the total volume of literature. We applied the exclusion criteria in several phases. In detail, files excluded both before the title-based and abstract-based screening, as well as after the full-text reading of the remaining literature.

The inclusion criteria included: (1) relevance of title, (2) evaluation of abstract and introduction for useful and relevant content, and (3) full-text reading of each article and publication. 

Exclusion criteria consisted of: (a) research papers, book chapters, and scientific articles without peer-review processes, (b) non-English-written articles or papers, (c) articles missing abstracts and introduction, (d) irrelevant publications, (e) articles and publications from bodies or organizations without a valid national or international status, as well as (f) unreferenced publications or unknown authors that were not members of relevant scientific communities. Criterion (d) refers to publications that seemed to be in-context but, after examination, we concluded were out-of-scope, e.g., a machine learning white paper named “machine learning for article analysis” that only contained promotional text for a paid tool without any technical information. Criterion (f) refers to publications that were not published in any scientific venue and are not referenced, cited or otherwise validated in any way from other technical whitepapers, reports or research publications. This essentially removes fake or plagiarized content from the survey and has no ties to whether a publication is included in paid venues. For example, papers from arxiv are included and criterion (f) does not apply to them.

### 2.2. Related Surveys 

Identifying misleading textual information mostly follows two different approaches: (1) the process of detecting falsehoods across multiple facets (e.g., multiple domains, languages, or sources), and (2) a process that pertains to only one facet. There are limited publications in the academic literature that compare methodologies focused on diverse aspects of deception detection of misinformation. Most surveys either tackle various methodologies that focus on the comparison of fake news detection on a single attribute, whether it will be language, a specific text domain or published by a specific source. This is highlighted in Zhou et al.’s survey regarding fake news [1]. The authors conducted a survey on the subject. In this article, we include some indicative surveys of single-dimensional methodologies although we opt to focus on cross-dimensional approaches. The topics covered include: (1) an introduction to the several definitions of fake news, (2) an analysis of the fundamental concepts regarding the analysis of fake news, such as style-based, propagation-based or user-based, and (3) a number of common lines of approach for detecting fake news, such as manual and automated fact-checking, stylistic analysis, etc. Their whole survey, however, focuses on single domain detection methods [1].

Zhang, X. and Ghorbani, A. [9] conducted an extensive survey about the methodologies addressed to online fake news detection. They clearly defined online fake news, along with its aspects, such as the news creator/spreader, news targets, news content or the social context. Moreover, they compared the existing methodologies and the datasets that are available for deception detection [9]. 

Shu et al. [9] focused on psychology and social theories related to the detection of fake news in social media. Their survey discovered the existing data mining algorithms that were being used. They also discussed the existing evaluation metrics and representative datasets. Finally, they stated the current open problems and future directions for the optimization of fake news detection in social media [10].

Bondielli et al. [11] conducted a survey about the existing techniques for the automatic detection of fake news and rumors. They focused on the extraction of fake news features and their categorization in two main groups: content-based and context-based approaches. Moreover, they collected approaches that were used for fake news detection and separated them into two categories: (1) techniques that faced the detection of fake news as a classification problem, and (2) those that aim to predict the class of the documents or assess their credibility. The former consisted of machine learning and deep learning methods, while the latter contained known pattern detection techniques, such as clustering and vector space models. All researched approaches that the authors examined used a specific dataset for both the training and validation phases and, thus, they conducted a single-domain evaluation of their models [11]. 

Oshikawa et al. [12] conducted a survey about multiple fake news detection methods and datasets. In their research, the focus was placed on natural language processing. The authors compared approaches based on the accuracy of their models. They conducted three tests separating the machine learning models according to the dataset used for training. Each of these three tests, however, used their allotted dataset for both training and testing (Single-Domain) [12].

Finally, Sharma et al. [13] conducted a survey for the existing methods for fake news detection. They separated the existing work into three groups: (1) content-based methods, (2) feedback-based methods, and (3) intervention-based solutions. The first category classified news according to the content of information. The second category classified the news based on the responses it received on social media, while the third one gave computational solutions for an active identification of fake news so as to protect users from exposure to false information. They also mentioned the advantages and limitations of each approach [13]. 

## 3. Background and Criteria

In this section, we introduce the generic workflow that Machine Learning (ML) approaches follow for the detection of misinformation along with their most used ML models. Moreover, we briefly present the metrics used for the performance evaluation of a model.

### 3.1. Machine Learning Models Used in Misinformation Detection

**Natural Language Processing (NLP)** is associated with the ability of machines to understand human language. When machines incorporate intelligence to their functions, they can perform various tasks, such as topic classification, translation, etc. [14]. **Machine Learning (ML)** provides models that are able to automate this process with accurate results [15]. Such models take advantage of two types of learning to set up: supervised and unsupervised learning. Additional or extended types of learning do exist; still, such models do not seem to have been given adequate focus for misinformation detection, and are thus not considered in the current study.

**Supervised learning** uses a set of labeled data. Such algorithms learn how to map specific inputs to appropriate outputs through guided information. Each label indicates the correct (or erroneous) answer that the model should predict (or not). The initial dataset (if there is only one) is usually split into training and testing data. The training set assists the model by “teaching it” patterns for prediction or classification, while the testing set is used to gauge its capacity in completing those tasks. The process is named “supervised learning” [16,17,18].

Regarding supervised machine learning models, this paper makes references to the following:

- Support Vector Machines (SVM): A model that utilizes training data to generate support vectors. These are used by the model to label the test data it is given [19].
- Random Forest: A classification and regression model that aggregates the results of multiple randomized decision trees [20].
- K-Nearest Neighbors (K-NN): A model that conceptualizes and plots the training data as a set of data points in a high dimensional space. It then categorizes each test point based on the K nearest data points in said high dimensional space [21].
- Logistic Regression: A linear regression model that uses a threshold and a sigmoid function to dichotomize and label the test data [22].
- AdaBoost: A model that mainly used in conjunction with short decision trees. AdaBoost takes multiple iterations of “weak learners” (i.e., decision trees), each with a different selection of weighted features, and aggregates them into one “strong learner” [23].
- XGBoost, LGBoost, Gradient Boosting: Machine learning models based on the Gradient Boosting framework [24].

**Unsupervised learning** bases the training on the experience gained from previous data handling. The training dataset is unlabeled and there is no guidance on the correct predictions. It allows the model to generate patterns for recognition on its own. This learning technique is used in more complicated tasks, such as misinformation detection (due to its large number of features) [15,16,17]. Regarding unsupervised machine learning models, this paper makes references to the following:

- Neural Networks: A collection of interconnected nodes (neurons), modelled to imitate neurons in a human brain. Layers upon layers of these units create bonds, which represent the underlying connections of the data in a set. This information is used to solve classification and regression problems [25].
- Long Short-Term Memory (LSTM): A variation of the Recurrent Neural Network (RNN), created to avoid the exploding and vanishing gradient problems found in standard RNNs [26].
- BERT Model: Bidirectional Encoder Representation and Transformers (BERT) is an NLP-based model, which trains such representations using unlabeled data. Post training can be fine-tuned to appropriate specifications, using labeled data [27].
- Convolutional Neural Network (CNN): A type of deep neural network that takes advantage of convolution (mathematic linear operation in matrices) [28].
- MMFD, SemSeq4FD: Custom frameworks created specifically for this problem set.

The following subsections present criteria used in this article to evaluate existing cross-* methodologies. The first group (Section 3.2) includes criteria (metrics) used for evaluating the ML models in various methodologies, the second group (Section 3.3) depicts criteria used for describing the data used in various phases of a methodology, while the third group (Section 3.4) refers to adaptability criteria, aiming to describe fake news from different domains, languages, or sources.

### 3.2. Model Evaluation Criteria

In Machine Learning, the detection of misinformation is a binary classification problem. This entails that there can be only two possible results, A or B. With regards to fake news detection, an article can be either real or fake. 

Table 2 presents the most used evaluation metrics in relevant methodologies. For their calculation, models utilize these basic metrics [6,29]. In context, the term “Positives” refers to actual Fake news, while the term “Negatives” refers to Real news. Adequate classification requires the use of more than one such metric to sufficiently report the performance of a model. 

Finally, there are mostly three common problems that a model may face during misinformation detection: (i) overfitting, (ii) vanishing gradient, and (iii) expanding gradient problems.

- The **Overfitting Problem** occurs when a model learns the training data points to such a degree, where it fails to adequately generalize and, thus, is unable to correctly classify any new information due to dissimilarity issues [30,31].
- The **Vanishing Gradient Problem** is found mainly in gradient focused machine learning models (e.g., Gradient Descent). This issue causes the model to stagnate since it cannot learn any further or has an extremely slow learning rate [32].
- The **Exploding Gradient Problem** is the opposite phenomenon of the Vanishing Gradient Problem. It occurs when long term ML components grow exponentially and cause the model to produce poor prediction results [32].

The above metrics are used as criteria to evaluate existing methodologies, along with two more attributes that we used during the following comparison of the cross-* approaches. 

### 3.3. Data Pre-Processing Criteria

Data pre-processing refers to the procedure used on a dataset to transform it into a more comprehensible format for the machine learning model. This includes adding, removing, splitting, and “cleaning” the data, in any way the developer of the model deems fit. Based on Castelo et al.’s [33] analysis, the features that accrue from this procedure can be split into four main categories (see Table 3). Features from each category are used in the training and testing phase of the machine learning models. They must be selected meticulously and deliberately so as to achieve a satisfactory result. Keep in mind that an increased number of features does not necessarily lead to better accuracy. These features appear in the examined approaches in Section 4.

### 3.4. Adaptability Criteria

These criteria reflect the extent to which a method can detect fake news from different domains, languages, or sources. Criteria synthesis is based on the classification criteria of Risk Assessment methods that were proposed by Gritzalis et al. [34]. 

The adaptability criteria utilize a three-level approach: (1) “Non-flexible”, (2) “Relatively flexible”, and (3) “Flexible”. Each level of adaptability refers to the capability of a method to successfully identify fake news in the cross-category it belongs. For instance, when a framework is characterized as non-flexible, it means that is more likely to identify misinformation on a single facet of the problem rather than in a cross-category case.

Our research team observed that insufficient information exists to support decision-making about the adaptability of fake news detection methodologies. To overcome this problem, we defined ad hoc parameters along with their respective value ranges that assist in determining the flexibility of deception detection methods. The parameters that we chose for the adaptability assessment of a methodology are: (1) ML model accuracy, (2) number of Sectors, (3) number of data pre-processing features, and (4) number of datasets. We selected the value ranges by observing the distribution of corresponding values that methodologies reach within the above-mentioned metrics. For instance, in Cross-Domain methodologies, the maximum number of covered domains in a methodology is eight. We consider this methodology as “Flexible”. The majority of the remaining Cross-Domain methodologies cover 4–6 domains. Thus, these methodologies were characterized as “Relatively flexible”, in comparison with those who cover eight domains. Finally, we concluded that if a method covers less than three domains, it is “Non-flexible”, since two domains provide limited adaptability. Below, we elaborate on the aforementioned parameters:

1. The first parameter is associated with the ML model accuracy of each approach. We utilize accuracy as it is the most commonly provided metric in the researched literature. When a methodology does not refer to this evaluation metric, it is assessed as “Non-flexible”. If this metric is calculated, then if its score is lower than 0.6, it is also “Non-flexible”, while if its score is between 0.6 and 0.8 the approach is “Relatively flexible”. Otherwise, it is “Flexible”.
2. The second parameter is related to the sector (domains/languages/sources) of each cross-category. Specifically, in Cross-Domain methodologies if an approach covers less than 3 domains then it is “Non-flexible”. In the case that the approach covers 3–7 domains, then it is “Relatively flexible”. Otherwise, it is “Flexible”. In Cross-Language methods, when an approach focuses on two languages, it is “Relatively flexible”, whereas when it focuses on three or more languages, it is “Flexible”. In the case of Cross-Source methodologies, if an approach uses data from less than 10 sources, it is “Non-flexible”. When it uses data from 10 to 100 sources, then it is “Relatively flexible”. Otherwise, it is “Flexible”. In the case of Multi-Cross methodologies, if the approach covers two cross-categories, it is “Relatively flexible”, otherwise it is “Flexible”. According to the categories to which this approach belongs, we separately calculate the adaptability of the related sectors. For instance, we consider a Multi-Cross methodology (A) that is Cross-Domain and Cross-Language. To characterize (A), we take into account the criteria related to Cross-Domain and Cross-Language methodologies, while ignoring Cross-Source-related criteria.
3. The third parameter refers to the data pre-processing features that the examined methodologies use. When an approach uses less than 5 features, it is “Non-flexible”. In case the approach uses 5–5 features, we also consider the ML model accuracy score. If it used more than 5 features and it achieves an accuracy score lower than 0.6, it is “Non-flexible”, otherwise when the accuracy score is equal or greater than 0.6, the methodology is “Relatively flexible”. Finally, if it used more than 15 features and achieves an ML model accuracy score more than 0.8, it is “Flexible”. Otherwise, if its accuracy score is equal or less than 0.8, it is “Relatively flexible”.
4. The fourth parameter is related to the datasets that each approach uses. Specifically, if the approach uses less than 5 datasets and covers less than 3 domains, then it is “Non-flexible”. If the datasets that are used cover 3 to 7 domains, the approach is “Relatively flexible”. Otherwise, it is “Flexible”.
5. The fifth parameter refers only to Multi-Cross methodologies. Our aim is to study whether including multiple cross categories refines the results of deception detection methodologies, thus increasing their flexibility. Specifically, if the approach incorporates two cross categories, then it is “Relatively flexible”. Otherwise, it is “Flexible”. Methodologies that use only one category are not considered as Multi-Cross.

To wholly evaluate an approach, we aggregated its respective flexibility parameters into a final flexibility score. We studied two scenarios: (1) the first four parameters for the assessment of Cross-Domain, Cross-Language, and Cross-Source methodologies, and (2) all five parameters for the Multi-Cross approaches. 

## 4. Cross-* Detection Methodologies

In this section we present datasets used by all detected methodologies during the training and testing phases. In addition, we divide all gathered cross-* detection methodologies into four main categories: (i) Cross-Domain Methodologies, (ii) Cross-Language Methodologies, (iii) Cross-Source Methodologies, and (iv) Multi-Cross Methodologies. Finally, for each category, we conduct a comparison of the including approaches. 

### 4.1. Datasets Used in Cross-* Methodologies

In this sub-section we introduce the most notable datasets used by the cross-* methodologies we surveyed. Datasets may include information from more than one domain. All presented datasets are publicly available, though a few of them may require some effort to be discovered.

**FakeNewsAMT** consists of 480 news texts, split into 240 real and 240 fake ones. The legitimate news articles are collected from six different domains: sports, business, entertainment, politics, technology, and education. The fake texts are generated and annotated through Amazon Mechanical Turk (AMT crowdsourcing marketplace) [35,36,37]. Freelancers receive the legitimate news and create corresponding fake texts of their choosing. The dataset contains claims, articles, and stories with their corresponding generated fake interpretations [35]. 

**Celebrity** is a dataset focused on news related to celebrities and public figures, such as actors, singers, socialites, and politicians. The main sources for the content found in this set are online tabloids and magazines (e.g., Entertainment Weekly, People Magazine, Radar Online etc.). It consists of 200 news articles, half of which are legitimate. Articles found in the dataset were verified using gossip-checking sites such as “GossipCop.com” [36].

**BuzzFeed-Webis Fake News Corpus 2016** consists of 1627 news articles sourced from 9 specific publishers in the week before the 2016 US Elections. Selected publications cover the entirety of the political spectrum. Specifically, 826 documents were acquired from mainstream sources, 256 left-wing and 545 right-wing. All issuers possess a blue checkmark on their Facebook Page, giving them a more trustworthy status towards readers [38].

**The Falsified and Legitimate Political News Database** is comprised of a total of 274 news articles about U.S. Politics. Half of them are legitimate. The rest are falsified versions of the same news documents [38].

**ISOT Fake News Dataset** (Univ. of Victoria Information Security and Object Technology) includes both real and fake news articles. Legitimate news was gathered from “Reuters.com”, while fake news articles were sourced from various publishers deemed unreliable by PolitiFact and Wikipedia. It covers a variety of topics, with the majority being political and world news. ISOT contains 21,417 real news articles and 23,481 fake ones [38].

**SVDC** (Syrian Violations Documentation Center) dataset is comprised of articles regarding the military conflict in Syria. Each document within the set is labelled by either 0 if it is fake or 1 if it is credible. True articles are confirmed considering the ground truth information acquired from the SVDC. It contains 805 articles, where 426 are true and the remaining 378 are false [38]. 

**US-Election2016** dataset consists of 691 articles. It contains 347 true and 344 fake articles. The dataset derives its information from news events around the time of the controversial 2016 United States Presidential Election. During this period, the term “fake news” revolutionized misinformation in all news and media outlets [33].

**PoliticalNews** contains a collection of 14,240 news pages, where the fake news was 7136 and true news was 7104. This dataset was produced from a collection of 1.6 million news pages, which were filtered to remove non-political pages. The final news pages come from 79 unreliable sites (based on Politifact, BuzzFeed and Opensources.co) and 58 reliable web pages [33]. 

**PolitiFact** is a relatively small dataset that contains 488 total claims. It is generated from claims and articles of the famous political fact-checking website “Politifact.com”. Its content revolves around politically themed articles and statements, involving subjects related to politics such as public policy. PolitiFact’s data are equally separated between truth and fake news [36].

**GossipCop** includes 8570 claims, collected from the tabloid and entertainment fact-checking website “GossipCop.com”. It contains data gathered from various media outlets. Data are scored on a scale from 0 to 10, depending on how fake they are. The scale goes from fake to real. Only 19% of all claims are fake, whereas the remaining 81% retain a score greater than 6 [36].

The **Chu et al. English Dataset** consists of 38,729 total news items that are written in English. Overall, 17,903 of them are fake, whereas the remaining 20,826 are real news. Each document is paired with titles, contents, subjects, dates, and labels. Each item is labelled using 0 if it is real and 1 if it is fake. Fake articles for the dataset were sourced from Kaggle and PolitiFact, whereas real documents were gathered from trusted news outlets, such as Reuters [37].

The **Chu et al. Chinese Dataset** contains a total of 38,471 news items, where 19,285 are fake and the remaining 19,186 are real. All articles are written in the Chinese language. Each news article is paired with an ID and labels regarding the veracity of the news. The false information in this dataset was sourced from the Weibo Community Management Center, while the real news items were crawled from Weibo and other trusted sources [37]. 

The **Rashkin et al. Dataset** is a PolitiFact-based dataset that consists of a total of 10,483 articles. Each article is graded on a 6-point truth scale (True—20%; Mostly True—21%; Half True—21%; Mostly False—14%; False—17%; Pants-on-Fire—7%). All information in this set was gathered from PolitiFact and spin-off sites, such as PunditFact [39]. 

**SLN (Satirical and Legitimate News)**, also known as Rubin et al.’s Dataset, is an English language Cross-Domain dataset. It contains 360 rumors/microblogs from various news sources, such as the Toronto Star, the NY Times, the Onion and Beaverton. The topics that are covered in this dataset are: (i) Civics (Gun Violence, Immigration, Elections), (ii) Science (Environment, Health, Other Sciences), (iii) Business (Finance, Tech, Corporate Announcements), and (iv) “Soft News” (Celebrity, Sports, Local News). Each topic is connected to 5 Canadian (Beaverton) and 5 American (the Onion) satirical pieces, as well as its corresponding legitimate news article [40].

**Fake or Real News (FRN)** is created by McIntire and is a manually collected dataset. It is comprised of 7800 (equally split) real and fake news articles. The fake information contained within the dataset was gathered from known unreliable media sources, whereas the truthful articles were acquired through trusted news outlets such as The New York Times, Bloomberg, and The Wall Street Journal. All data were gathered from 2015 to 2016 [41].

**Kaggle-JR (KJR)** is composed of 4000 news, where 1600 are fake and 2400 are real. Fake news was scraped from unreliable news sources such as Before It’s News, Daily Buzz Live, and Activist Post. Real news was gathered from BBC, Reuters, CNN, ABC News, and The New York Times. All data were collected from September 2017 to October 2018 [41].

**NELA-GT-2018** consists of 713,000 articles gathered from over 194 news media outlets. The dataset is already pre-labelled with annotations regarding veracity, bias and conspiracy. Many of the unreliable media sources, that were used in the data gathering, copied news content from the reliable sources. Hence, the dataset contained many duplicate stories that had to be filtered out [41].

**LIAR** contains 12,800 manually labelled short statements from the “Politifact.com”. Its contents consist of a mixture of democratic and republican statements as well as a copious amount of social media posts. Each claim has been labelled based on its truthfulness. The six labels used in claim tagging are: (i) pants-fire, (ii) false, (iii) barely true, (iv) half-true, (v) mostly true, and (vi) true [42,43].

**Weibo** is currently one of the largest micro-blogging social networks in China. Users enjoy an operation, named comment-only, that permits them to give feedback on a post without forwarding [44,45,46,47]. It consists of 2313 rumors and 2351 non-rumors. The data was collected via the Weibo API and annotated by the Sina community management center. Weibo provides the original posts and messages, along with their corresponding reposts and replies [44].

**LUN** is split into two separate sub-datasets, LUN-train and LUN-test. The training set contains 24,000 articles from the Onion and Gigaword excluding Associated Press WorldStream (APW) and Washington Post/Bloomberg Newswire service (WPB) sources. The testing set consists of 1500 news documents, solely from APW and WPB sources. LUN is a Cross-Source dataset since its training and test subset do not share the same fake and real news sources [6].

**RCED** incorporates two datasets into one: Ma et al.’s set [45] as well as Song et al.’s Microblog set [45]. It contains 2955 true and false news in the Chinese language. RCED is mostly used for rumor detection. It obtains its information from Sina Weibo and filters out documents with two sentences or less [6].

**Jeronimo et al.’s Dataset** comprises 207,914 articles gathered from 2014 to 2017. It covers the domains of Politics (55,403), Sports (63,600), Economy (50,924), and Culture (37,987). All data were collected from to Brazilian fact-checking services: e-Farsas and Boatos [46].

**FakeCovid** consists of 5182 fact-checked articles for COVID-19, collected from Poynter and Snopes. It contains articles that were selected from 4 January 2020 to 15 May 2020. It is a multilingual Cross-Domain dataset since its information is collected from 92 fact-checking websites and is written in 40 languages. Each article is annotated into 11 different categories, based on its content [48].

### 4.2. Cross-Domain Methodologies

#### 4.2.1. Cross-Domain Methodologies Analysis

This category includes the methodologies and frameworks that focus on detecting fake news from different domains. A domain, in this context, is a specific topic. Each subject uses distinct vocabulary. The most common domains, used in the following approaches, are politics, celebrity news, education, sports, entertainment, and technology.

Pérez-Rosas et al. [47] developed a methodology for the automatic authentication of fake content in online news. The authors used two datasets, FakeNewsAMT and Celebrity, for the construction of a deception detection model. Their work concentrated on a set of linguistic features over a linear Support-Vector Machine (SVM) classifier along with five-fold cross-validation. They evaluated their approach by calculating the model’s accuracy, precision, recall, and F1-score while testing the hypothesis that a greater amount of training data improves the detection of fake content. They also elaborated on which features had the best performance for each dataset for Cross-Domain analysis by using the best three feature sets. Their work detected that some domains such as entertainment, business, and sports, contain more domain-dependent vocabulary than others. 

Gautam A. et al. [49] presented a fake news detection system that explored whether a news article is authentic. They used two publicly available datasets; FakeNewsAMT and Celebrity. Their approach exploited three tools: (1) Spinbot, which checked for paraphrasing; (2) Grammarly, which conducted grammar-checking; and (3) GloVe, which was responsible for word embedding. The authors applied a 5-fold cross-validation to develop their Random Forest (RF) classifier. The RF Model used labeled data to predict the authenticity of news in the training phase. 

In order to evaluate the proposed methodology, the authors conducted a Cross-Domain analysis. Additionally, they examined whether the amount of training data affects the performance of the proposed model. Finally, they assessed the performance of a multi-domain trained approach.

The authors concluded that when their model had a greater amount of training data, its performance was optimized. Moreover, the fact that they reached 70% accuracy with their model with the Celebrity dataset, with it being tested on six completely different domains, seems promising.

Saikh T. et al. [35] proposed two slightly different models (Model 1 and Model 2) for the detection of fake news in online news contents. Both models were based on deep Learning and aimed to solve the problem of multi-domain detection. The authors used the FakeNewsAMT and Celebrity datasets.

Model 1 and Model 2 had the same layered architecture: (A) Embedding, (B) Encoding (Bi-GRU), (C) Word level Attention, and (D) Multi-layer Perceptron (MLP). The difference lies in the approach they used in the first layer of each model. In Model 1, the authors used the pre-trained FastText model to embed each word, while in Model 2, the Embedding for Language Model (ELMo) method was used.

They conducted four types of experiments: (1) a multi-domain analysis, (2) a Cross-Domain analysis, (3) a multi-domain training, along with domain-wised testing, and (4) a domain-wise training, along with a domain-wise testing. 

It is noteworthy that the ELMo model, which is used in the first layer of Model 2, produced better results in all experiments. This was expected to occur since ELMo has shown that it performs better in multi-domain datasets. They also observed that the domains of entertainment and sports have diverse linguistic properties, writing style, and vocabularies. On the contrary, the domain of education has linguistic properties and vocabulary that are similar to those of technology, business, or politics.

Castelo S. et al. [33] introduced a topic-agnostic (TAG) classification approach that focused on the identification of fake news pages. The model received a web page as its input and examined whether this page was likely to contain fake news. Their research included all types of active misinformation. They used three datasets: Celebrity, US-Election2016, and Political News. As for their experimental setup, the authors used: (1) the NLTK library to compute Morphological Features, (2) the Linguistic Inquiry and Word Count for Psychological Features, (3) Textstat for Readability Features, as well as (4) BeautifulSoup and Newspaper for web-markup features. 

The performance evaluation of the model consisted of three scenarios: (1) training with several combinations of feature sets, (2) examination of the model’s behavior over time, and (3) examination of effectiveness in Cross-Domain datasets. The authors observed that a content-based model is capable of learning to detect fake news for specific topics in a specific period of time. The authors limited this part of the research to the domain of politics. 

Lin J. et al. [50] presented a framework that extracted 134 features and built traditional machine learning models, such as XGBoost and Random Forest. They also developed a deep learning-based model, the LSTM with a self-attention mechanism. Their main dataset was the FakeNewsNet, which contained several types of information, such as news content, social context, and spatiotemporal information. The labels were obtained from the Politifact and Gossip Cop fact-checking websites. The framework included: (1) preprocessing, (2) feature extraction, (3) machine learning, and (4) deep learning. 

In the first group of their experiments, they evaluated the performance of their framework in two scenarios: (1) without hyperparameter tuning and (2) with hyperparameter tuning. The second group included all the above models of the approach, but they used three datasets: Politifact, Gossip Cop, and the previous two combined. 

The authors proved that their models can successfully detect fake news when they deal with complex and Cross-Domain articles. Their framework reached an accuracy consistently greater than 80% despite any hyperparameter tuning modifications. It is significant that the authors came up with these result by only focusing on the text information of a news article, without taking into consideration other information, such as social media content or spatiotemporal data. 

Kula et al. [51] created a text analysis/fake news detection framework based on a recurrent neural network (RNN). While it is not explicitly stated in the literature, this methodology was indeed Cross-Domain. This was, in part, due to the training process the neural network underwent.

The pre-processing stage was based on natural language processing. Specifically, the authors utilized the Flair library to execute text pre-processing, as well as the “glove” method to create word embeddings. For text classification, Kula et al. utilized RNN, with a focus on Gated Recurrent Units (GRU) and LSTM. They trained the model with two different datasets. The first (single-domain) was trained using only the ISOT dataset, whereas the second (Cross-Domain) was trained with a combination of the ISOT and a Kaggle dataset containing mostly fake news. 

This approach, utilizing the Flair Library, the “glove” method for word embedding in conjunction with the RNN, provided Precision, Recall, and Accuracy scores of 0.9982, 0.9991, and 99.86%, respectively. These scores show an unusually high level of success. Without understating the possibly groundbreaking work performed in this research, such values for accuracy, precision, and recall metrics tend to be associated with overfitting. The literature does not address this prospect and, therefore, we cannot add it as the best score in our comparison presented in Section 5.

Shu et al. [52] presented a framework for Twitter fake news detection, built on Hierarchical Propagation Networks. Their model attempted to recognize potential fake news by analyzing two propagation levels: (1) macro, which includes behavioral patterns, connections of users through retweets, etc., and (2) micro, which consists of individual user interactions, replies, likes, etc.

The authors used three datasets; FakeNewsNet, PolitiFact, and GossipCop. In the pre-processing stage, they analyzed them based on three different manners:

- **Structural**: Determination of structural patterns in how fake news spreads globally (macro-level) and identification of structural patterns in conversations, where viewpoints are expressed (micro-level).
- **Temporal**: Recognition of opinions and emotions through the time period and frequency in which a conversation or a response took place.
- **Linguistic**: Analysis of textual information, such as emotional language, specific vocabulary, and syntax.

This framework contains pre-made machine learning models, such as Gaussian Naïve Bayes, Decision Tree, Logistic Regression, and Random Forest. The authors tested their method in two ways: (1) each level of analysis separately (e.g., only Temporal Analysis or only Linguistic Analysis), and (2) a combination of analysis levels. The method provides scores of 0.852, 0.844, 0.860, and 0.852 for Accuracy, Precision, Recall, and F1, respectively.

#### 4.2.2. Comparison of Cross-Domain Methodologies

In this section, we perform a comparison of the aforementioned methodologies. Table 4 presents the performance evaluation of the Cross-Domain methodologies. Since the necessary values were not available in the data in some cases, we annotated them as N/A (Not Available). Table 4 corresponds to a summary of results and their cross-comparison to extract valuable information on which methodology works best under which circumstances. Table 5 presents the features that each approach uses for the data pre-processing stage. Finally, Table 6 depicts the domains that each methodology covers, whereas Table 7 presents the adaptability level of each approach. In Table 5 and Table 6, empty slots correspond to unavailability of a feature in the corresponding methodology.

Kula et al. and Shu et al.’s research papers are not contained in Table 5 and Table 7, due to the fact that they do not explicitly state the data pre-processing features used in their respective methodologies. Instead, they make use of custom data pre-processing features to achieve their scores. Thus, we cannot state which features are utilized and are unable to accurately gauge the flexibility of these approaches.

Through analysis of the Table 4, Table 5, Table 6 and Table 7, we can deduce the following. By comparing the highest (Lin J. et al. XGBoost) with the lowest (Castello et al.’s Random Forest) Accuracy scores, we notice a nearly 0.24-point drop. This is unexpected as they test many common machine learning techniques including the Random Forest, which performed the worst. Moreover, we notice similar feature usage from both approaches. Specifically, N-grams, word count, as well as psychological features are common between the two methods. The dataset topics being used are also identical (Political and Celebrity). 

Strictly based on performance metrics, Kula et al.’s approach seems to be the best, as it produces the highest results in terms of accuracy, precision, recall and F1-Score. As stated previously, however, these scores may be associated with overfitting. Such an issue is not referenced in their literature; thus, reproducing this approach would be the sole means of confirming its performance. Therefore, we would also have to include Shu et al.’s approach, as it has the second-best performing methodology, and its results are within the current margin of realistic performance.

Delving deeper into the parameters of these models, we notice the following: PolitiFact contains general Political and Public policy News concerning various individuals and events, whereas the US Election 2016 dataset focuses on a specific time period of the political news scene. Moreover, the other models that share common datasets provide rather consistent results (excluding Saikh et al.’s ELMo model). Thus, we can hypothesize that, regarding Cross-Domain deception detection, it is more effective to use datasets that cover less topics with more events. It is also important that we use Accuracy as the main metric of comparison, due to the lack of F1-Scores.

### 4.3. Cross-Language Methodologies

#### 4.3.1. Cross-Language Methodology Analysis

This category includes methodologies and frameworks that can detect fake news from different languages (e.g., training in English and testing in Chinese).

Chu S. et al. [37] introduced a study that examined different features between two languages. Specifically, they chose English as a “simple” language and Chinese as a more “complex” one. They extracted the key features for both languages and performed classifications based on the Bidirectional Encoder Representations from Transformers (BERT) model, since it often has better performance in textual classification tasks. In order to validate their idea, they calculated the similarity between the keyword features of these languages, using TextRank, a graph-based text ranking model. 

For their experimentation, the authors evaluated four scenarios: (1) English training data and English testing data, (2) English training data and Chinese testing data, (3) Chinese training data and Chinese testing data, and (4) Chinese training data and English testing data. For each scenario, the metrics of accuracy, precision, recall, and F1 were calculated. Finally, they examined the similarity between the Chinese and English keyword features. After their experimentation, the authors concluded that it is preferable for a model that addresses Cross-Language fake news detection to be trained on languages with more complex keyword features. During testing, the researchers observed that results for both the Chinese-English and English-Chinese models did not deviate significantly from 55% accuracy. It was apparent when they removed keywords that were similar in both languages. This leads us to believe that the model was practically selecting at random. Perhaps this model would function better if the languages used originate from the same language family (e.g., Italian and Spanish).

Vogel I. et al. [53] created three machine learning approaches capable of recognizing falsehoods in both English and Spanish. Initially, they tested two models to establish a solid baseline. The first used ELMo sentence representations in conjunction with a CNN, while the second was a standard SVM with Term Frequency-Inverse Document Frequency (TF-IDF) and N-Grams for the data-preprocessing. For their first approach, they focused on words that were associated with emotions. The model consisted of a SVM combined with TF-IDF. The second approach combined SVM and Logistic Regression, where they focused on textual cues such as emojis, uppercase letters, and phrases. Finally, in the third approach, the authors performed hyperparameter tuning to effectively combine SVM and Logistic Regression from the previous approach. In this approach, the performance metrics were far more stable than those found in Chu et al. This could be due to the focus on hyperparameter tuning and data pre-processing. Additionally, we notice that the tests were conducted on relatively closely related languages (English and Spanish).

Abonizio H. et al. [54] created a method for detecting fake news across three languages: English, Spanish, and Portuguese. In order to convert all textual information into homogenous text without non-textual elements, the authors performed the following tasks: (1) cleaning, (2) filtering, and (3) noise removal. Textual data was converted into features, which were segregated into three categories: (i) Complexity, (ii) Stylometric, and (iii) Psychological. The authors evaluated their method by using k-Nearest Neighbor, SVM, Random Forest, and Extreme Gradient Boosting. Finally, the testing was conducted using k-fold cross-validation. 

Similarly, Vogel et al.’s performance metrics showed a higher grade of stability and accuracy in recognizing fake news and satire. Moreover, the languages tested in this paper were closely related (English, Spanish, Portuguese). English, Spanish and Portuguese are all Latin languages with similar alphabets and correlations in their grammar, as opposed to e.g., German (which is an Indo-European language with more differences), or Greek (which has a different alphabet and grammar altogether).

Guibon G. et al. [55] presented an automated fake news detector, which can handle deception recognition in multiple languages. They focused on English and French. The dataset used for training and testing was provided during a hackathon in which the research team took part. It contained a total of 6358 texts, gathered from a variety of websites, including YouTube French transcripts (closed captions). They aimed to classify the dataset’s content into three categories: Fake News, Trusted, and Satire. For data representation, they utilized four separate lines of approach as well as combinations of those. Specifically, the authors chose TF-IDF, FastText, Word2Vec, and Hashing Trick. This was undertaken to achieve a classifier that can better generalize its predictions. For the classifiers, the researchers initially used a simple Decision Tree, an SVM template from a hackathon, and their own custom Light Gradient Boosting Machine. Once a baseline was established, they attempted to optimize these approaches, in addition to creating a custom Convolutional Neural Network with two hidden layers. 

#### 4.3.2. Cross-Language Methodologies Comparison

In this section, we conduct a comparison of the above-mentioned Cross-Language methodologies. Table 8 presents the performance evaluation of the Cross-Language methodologies. Since some required information was not available, relevant slots in tables are set to N/A (Not Available). Grey highlights in the table depict the evaluation metrics with the best performance per work. Table 9 presents the features that each approach uses for the data pre-processing stage. Finally, Table 10 depicts the languages that each method covers, whereas Table 11 presents the adaptability level of each approach.

According to Table 8, Table 9, Table 10 and Table 11, we notice that language similarity is important during Cross Language deception detection. The relation between English and Spanish, as well as English and French, assist in the production of higher scores across the board (e.g., Guibon et al. and Abonizio et al.).

The approaches of Chu et al. and Vogel et al. provide similar results (F1-Scores of 0.74 and 0.715, respectively), despite the vast difference in feature selection. This leads us to believe that in Cross-Language fake news detection, the model that is used or created for the task is the most important segment of the equation. In this regard, Guibon et al.’s Optimized SVM seems to perform the best. However, the overall results of the aforementioned Cross-Language approaches provide slightly inferior results when compared to the Cross-Domain frameworks. The average F1-Score for the Cross-Language approaches is 0.7727 whereas that of the Cross-Domain approaches is 0.8085 (not including Kula et al.’s results for reasons previously stated).

### 4.4. Cross-Source Methodologies

#### 4.4.1. Cross-Source Methodologies Analysis

This category focuses on the detection of fake news among news that originates from different sources. 

Asr T. et al. [56] faced two challenges in fake news detection: source reputation and content veracity. The authors used two datasets that consisted of real news articles that were found by the Buzzfeed and Snopes fact-checking websites, along with Rashkin et al.’s and Rubin et al.’s datasets. They randomly selected 312 of the 4000 news articles and assessed them manually. The datasets used for testing the model contained distributions of Satirical, Hoax, Propagandistic, and trusted news content, which varied drastically.

The work used individually assigned veracity labels that were indicative of misinformative content. The authors examined whether reputation-based and individually assessed news articles were distinct. The veracity labeling system included the following labels: (1) false, (2) mixture of true and false, (3) mostly false, (4) mostly true, and (5) true. As for the sources, they used the categories of: (i) Ambiguous source, (ii) Context source, (iii) Debunking source, (iv) Irrelevant source, and (v) Supporting source.

The fact that the datasets consisted of several news contents could potentially cause the model to perform sub-optimally. While the results were encouraging for the Rashkin et al. dataset, the model’s performance decreased significantly when tested on the data provided by Rubin et al. A possible solution would be to pair the training and testing sets based on the distribution of content.

Huang Y. H. et al. [41] focused on the phenomenon of Cross-Source failure. They built a model that generalized the existing content-based methods. Their aim was for the model to perform consistently when fake news was coming from unknown media sources. Various publishers and writers tend to have different writing styles. Thus, each source may focus on various aspects of the text. The authors presented a framework that consisted of two steps: (1) the syntactic pattern construction and (2) feature debiasing. For their experiments, they used three well-known datasets: Fake or Real News (FRN), Kaggle-JR (KJR), and NELA-GT-2018 (NELA). 

For their Cross-Source validation, the authors chose not to test different datasets but to split the datasets according to their media sources. They used 80% of the sources for the training phase, while the other 20% were used in the testing phase. The former were called seen sources, and the latter were referred to as unseen sources. 

When we reviewed the performance of this methodology, we observed the following. During testing, the performance metrics were significantly higher (as expected), since the model was trained and tested with the same dataset. When the training and testing sets differed, there was a drop in performance across the board, excluding the combination NELA-KJR with a SVM (Support Vector Machine). This model—dataset pairing retained 0.825 Accuracy. 

Karimi H. et al. [57] introduced a Multi-source Multi-class Fake news Detection (MMFD) framework. They focused on fake news from multiple sources since they provide rich contextual information about fake news and offer unprecedented opportunities for advanced fake news detection. They combined information from multiple sources and examined different degrees of fakeness. The proposed model consisted of three components: (1) automated feature extraction, (2) multi-source fusion, and (3) automated degrees of fakeness detection. The authors used the LIAR dataset. Since LIAR consists of three sources, the authors decided to add another type of source. Finally, the sources that were used for the evaluation of the framework were: (1) statements, (2) metadata, (3) history, and (4) reports.

Their research and testing suggest that adding multiple sources to training and testing may be effective in increasing a model’s performance. This is, however, especially true, for the researchers’ custom model, due to it containing a discriminative function (referred to as MDF).

Wang L. et al. [58] presented a framework for the Cross-Domain learning of propaganda classification. The framework performed the tasks of: (1) data collection, (2) feature selection, (3) training procedures, and (4) different learning methods and analysis. They designed features and constructed classifiers for propaganda labeling with the use of Cross-Domain learning. They used five datasets from three different domains to explore potential characteristics for propaganda detection. The datasets consisted of speeches, news, and tweets. The authors utilized different machine learning methods including LSTMR, an improved version of LSTM. The authors combined datasets during the training phase of the algorithm. During experimentation, the authors evaluated their approach in the scenarios of in-domain and Cross-Domain performance. Experiments included calculations for Precision, Recall, and F1 scores. 

The benefit of this approach is that the authors used LSTMR, which mitigates the problem of overfitting and enhances the Cross-Domain detection of fake news. They also evaluated the performance of their model by combining the three datasets in the training phase. Unfortunately, due to the heterogenous sources of data, the performance was not improved.

#### 4.4.2. Cross-Source Methodologies Comparison

In this section, we compare the aforementioned methodologies. Table 12 presents the performance evaluation of the Cross-Source methodologies. We highlight the best scores for each metric. Table 13 gathers the features that each approach uses for the data pre-processing stage. Finally, Table 14 presents the adaptability level of each approach. By examining Table 12, Table 13 and Table 14 and comparing the methods with the best (Asr et al.’s SVM) and worst (Wang L. et al.’s LR) F1-Scores, we deduce that feature selection may be the most important task for Cross-Source deception detection. The difference between the two methods’ F1-score is nearly 0.47. Additionally, they both utilize SVM as their classification model of choice. Another reason for the better performance is due to the fact that SVM models require large amounts of data for training. Asr et al. trained with a large custom set of four (4) datasets, whereas Wang L. et al. used a smaller custom set of speeches, news, and tweets. Moreover, we notice a large discrepancy in feature selection. Asr et al. used a large number of different features in comparison to the study of Wang et al., which only added Web Markup Words. More specifically, we notice that Asr et al. focused on readability and psychological features. This seems to be a logical decision, due to the fact that the focus in Cross-Source deception detection lies within the reaction the writer is attempting to invoke. Centering the attention on emotional vocabulary and thought-provoking punctuation marks (exclamation points, question marks, ellipses, etc.) provides better results.

### 4.5. Multiple-Cross Methodologies

#### 4.5.1. Multiple-Cross Methodologies Analysis

In this category, the methods and frameworks that combine more that one of the previous categories are presented. We refer to these approaches as “multi-cross” to indicate the interpolation of various characteristics and training models from numerous types of cross-* methodologies.

Y. Wang et al. [6] demonstrated an end-to-end model, SemSeq4FD, that makes early fake news detection based on enhanced text representations. They addressed the problem of fake news detection in the following way: they assumed that fake news detection is a binary classification problem. The authors created a framework that included three modules: (1) Sentence Encoding, (2) Sentence Representation, and (3) Document Representation. For their experiments, they used the LUN and SLN datasets that have English-based content, as well as Weibo and RCED which contain news in Chinese. They tested their model using 7 state-of-the-art models that they separated in three main categories: (1) Machine learning models included SVM and Logistic Regression. (2) Non-graph deep learning network models consisted of Convolutional Neural Network (CNN) and BERT. (3) Graph-based deep learning network models contained Graph Convolutional Network (GCN), Graph Attention Network (GAT), and Graph Attention Network with two attention heads (GAT2H). 

This model seems to produce the best results in its category, regarding metrics such as Precision, Recall and F1. The results for in-domain testing are, as expected, higher than the Cross-Domain performance; however, the researchers managed to achieve a higher stability than most other models. This could be due to the researchers training the model on text representations of increasingly higher levels of abstraction (word-level, sentence-level, and document-level).

Jeronimo C. et al. [46] introduced a methodology for the extraction of subjectivity features of real and fake news. They used a collection of subjectivity lexicons built by Brazilian linguists. They used a large-scale dataset from two Brazilian media platforms: Fohla de Sao Paulo and Estadao. 

Their method was evaluated based on five scenarios: (1) The legitimate vs. fact-checked fake news scenario—both legitimate and fake news as training data, regardless of their domains and source. (2) The Cross-Domain scenario—training data from a specific domain and testing data from a domain that was not included in the training. (3) The Cross-Source scenario—training with legitimate data from a specific source and testing with data from another source. (4) The satire as fake news surrogate scenario—the training set contained satires instead of fact-checked fake news, while the testing set used fact-checked fake news. (5) The satire for fake news augmentation scenario—a mix of fact-checked fake news and satires was used in the training set, and fact-checked fake news was used in the testing set. Finally, they used XGBoost and Random Forests as classification learning methods since they are characterized by their strong predictive power and are appropriate for complex domains. In this methodology, the researchers attempted to approach the issue of deception detection in a completely opposite manner than most. Where many would abstract the dataset to provide a topic agnostic vision to the model, Jeronimo et al. focused on the language that adds to this property (subjective language). Through testing, this model’s accuracy is relatively high; however, we cannot come to a clear conclusion without the remaining metrics (Precision, Recall, and F1).

Shahi, G. and Nandini, D. [49] built and presented an opensource multilingual Cross-Domain dataset that contained fact-checked news articles for COVID-19. They also constructed a classifier that helped the detection of fake news in the time of the pandemic. Their aim was to develop a machine-learning-based classifier to detect any misinformation occurring at the time of pandemic. They built the dataset based on the Snopes and Poynter fact-checking websites. In order to construct their dataset, the authors followed the steps of: (1) data collection, (2) data annotation, (3) data cleaning and preprocessing, (4) data exploration, and (5) classification.

#### 4.5.2. Comparison of Multi-Cross Methodologies

In this section we perform a comparison of the presented Multi-Cross methodologies. Table 15 presents the performance evaluation of the Multi-Cross methodologies. Since the values of the data needed were not available in some cases, we annotated them with N/A (Not Available). Table 16 concentrates the features that each approach uses for the data pre-processing. Table 17 depicts the domains that each methodology covers. Finally, Table 18 presents the adaptability level of each approach, whereas Table 19 depicts the categories to which each Multi-Cross methodology belongs.

Through analysis of the statistical information stated in Table 15, Table 16 and Table 17, we produce intriguing findings. On the one hand, Jeronimo et al.’s Random Forest generates the best accuracy and is quite consistent with all the models tested. However, we are not provided with an F1-Score. Their approach is assessed as “Relatively flexible” since it is the only methodology that covers two cross categories, as depicted in Table 16. On the other hand, Y. Wang et al.’s model, SemSeq4FD, seems to be more effective in misinformation detection. In detail, their model reached the highest Precision, Recall, and F1 scores (0.8900, 0.8800, and 0.8967, respectively). It is assessed as “Flexible” because their methodology covers eight domains, thus providing greater flexibility compared to the rest of the Multi-Cross methodologies.

Finally, we notice that datasets used are reasonably sized and feature use is not excessive. Therefore, we can conclude that, the most important factor in Multi-Cross fake news detection, is to find the balance between datasets, models and features used.

### 4.6. Other Cross-* Datasets

A few additional important datasets, which fall into one or more cross-* categories, are also introduced. We include them in our study since we wish to assist in furthering the field of fake news detection. Hence, our aim is to provide as many tools as possible.

**FEVER** consists of 185,445 claims. The annotators created both legitimate and fake claims based on information extracted from selected Wikipedia pages regarding topics such as Science and Education (Cross-Domain). These claims were labelled as: (i) supported, (ii) refuted, or (iii) not enough info. For the first two tags, the annotators recorded the sentences that were necessary to form their decision. Data validation was conducted in three different forms: 5-way inter-annotator agreement, agreement against super-annotators, and manual validation by the authors [60].

**FacebookHoax** is constructed from data collected using the Facebook Graph API. It contains public posts and their corresponding likes from a manually selected list of Facebook pages (Cross-Source). FacebookHoax is composed of 15,500 posts from 32 different pages, where 14 of them contain conspiracy-related information and 18 contain scientific information. The user interaction amounts to nearly 2.3 million likes by 900k separate users. Overall, 57.6% of the content of the dataset comprises hoaxes, while the remaining 42.4% is made up of non-hoaxes [60].

**Twitter** is a microblog dataset collected from “Twitter.com”. It contains a total of 778 tweets confirmed by snopes.com. Overall, 64% of them are rumors, while the rest are non-rumors. These events were reported in a 10-month timespan in 2015 and covered topics such as politics and public policy (Cross-Domain) [44].

**Genes-Kaggle** is an independent dataset created by Yunus Genes. It comprises more than 52,000 articles from large news agencies. Specifically, 29,000 of them were collected from The Guardian and 12,000 from the New York Times (Cross-Source). Moreover, 12,000 articles were labelled as fake. The dataset covers a multitude of topics, including US News, Politics, Business, World News (Cross-Domain) [13].

**SemEval-2016 Task 6** contains 4870 Tweets, created as training data for machine learning assisted stance detection. It consists of claims regarding topics such as Atheism, Climate change, and Hillary Clinton (Cross-Domain). Each row consists of the tweet in question and a manually annotated tag, which represents the target of the claim. Overall, 25.8% of the tweets are in favor of the target, and 47.9% are against, whereas 26.3% have a neutral stance [6].

**BuzzFeedPolitical** contains 120 articles published during the 2016 U.S. Presidential Elections. Its content is equally split into real and fake news. Each article is manually annotated as either Real or Fake, depending on the source that published it (Cross-Source). Sources were cross-referenced with a list that contained known trustworthy and fake news agencies [60].

**Political-1** includes 225 political articles, equally divided in three categories: Real, Fake, and Satire. Each story was collected from well-known sources pertaining to each category. Fake stories were cross-referenced using Zimdars’ list of fake and misleading websites. Additionally, trustworthy articles were sourced from Business Insider’s Most trusted list. Finally, satirical texts were scraped from websites that explicitly state that their content has no intention of misleading the reader (Cross-Source) [60].

**BuzzFeedNews** consists of 2285 claims and articles based on the “Politifact Fake News Almanac”. It contains posts that were considered the biggest fake news hits on Facebook in 2017. It contains political data which are labelled on a 4-point scale: Mostly True, Mixture, Mostly False and No Factual Content [61].

**BS-Detector** is a browser extension that was developed for the veracity checking of fake news. It scrapes all links on a webpage for references to non-trustworthy sources. These links are then cross-referenced with a manually compiled list of fake news domains. The labels come up as the output of the extension, and thus, there is no need for human annotators. These labels can be either Reliable or Unreliable [62].

**CREDBANK** is the largest current existing dataset. It includes 60 million tweets spanning across the year 2014 and divided into 1049 separate events (Cross-Domain). The dataset falls within the domain of credibility assessment. It uses thirty different annotators to label each tweet’s credibility. The dataset consists of 24% non-credible data, while the remaining 76% is credible [63,64].

**PHEME** contains a collection of 6435 Twitter rumors and non-rumors posted during breaking news. These tweets are related to the following events (Cross-Domain): Charlie Hebdo, Sydney Siege, Ferguson, Ottawa Shooting, GermanWings Crash, Putin Missing, Prince Toronto, Gurlitt, and Ebola-Essien [65]. Each rumor is labelled as either True, False, or Unverified. Non-rumors comprise 64% of the contents of the dataset, whereas rumors are split as follows: 16% are True, 10% are False, and the remaining 10% are Unverified [64].

**FNC-1** was generated for the FakeNewsChallenge (FNC), a competition organized to explore how artificial intelligence technologies could be leveraged to combat fake news. The dataset contains 49,972 news articles that are annotated as follows: Unrelated (73.1%), Discuss (17.9%), Agree (7.3%), and Disagree (1.7%) [66].

### 4.7. Tabulated Processed Information

At this point, we have presented the majority of the important information needed to analyze these methodologies. In order to provide further information on this matter, we have developed and added an Appendix at the end of this paper. The Appendix A contains the following information:

- Cross category of the methodology.
- Sectors of the cross category which are contained in the dataset used.
- The data pre-processing applied to each dataset.
- The type of representation used for training.
- Machine learning type.
- Metrics for which results are provided.

## 5. Discussion on Cross-* Methodologies

**Figure 2:**
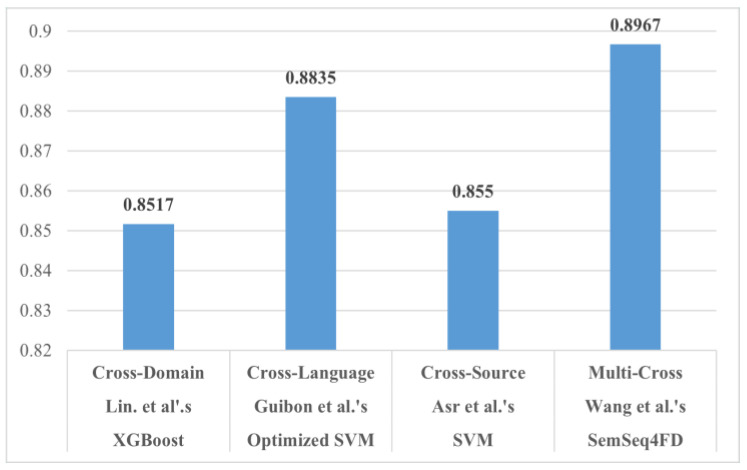
Best F1-Score Performance per Cross-Category.

This section explains the results of the comparison that we conducted among the four cross-categories. Figure 2 depicts, for each cross-category, the approach that achieved the best performance regarding the F1-Score and the machine learning technique that the authors used. In detail, the Cross-Domain approach of Lin et al., using the XGBoost model, reached 0.8517. For the Cross-Language approach of Guibon et al., using an optimized SVM model, a score of 0.8835 was reached. In the Cross-Language approach of Asr et al., a score of 0.8550 was achieved using an SVM model. Finally, among the Multi-Cross methodologies, Wang et al., with their SemSeq4FD model, reached the highest score out of all the cross-categories—0.8967.

**Figure 3:**
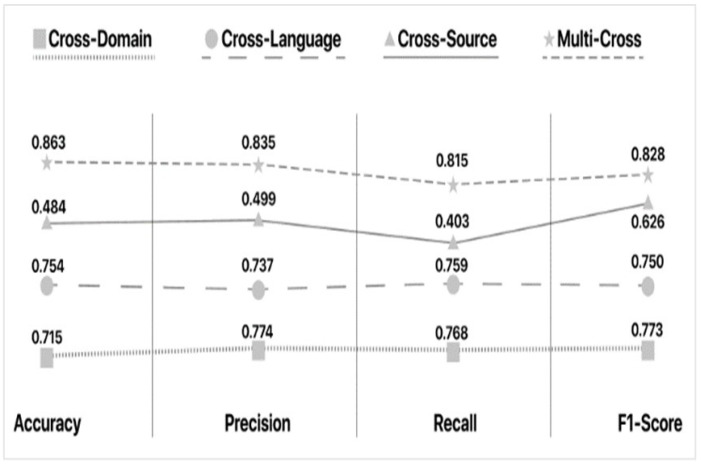
Average performance per Cross-Category.

Figure 3 depicts the average performance of the four cross-categories regarding the metrics of Accuracy, Precision, Recall, and F1-Score. For the calculation, we considered only those approaches that included such information. We can observe that the highest score in all evaluation metrics belongs to the Multi-Cross category. These methods seem to combine the advantages of 2–3 cross-categories. For Multi-Cross methods that utilize different domains (e.g., politics, education, celebrity news, etc.) containing dedicated vocabularies, we see that these models gain the ability to detect fake news across several topics without much tradeoff in accuracy.

In cases in which a framework combines different languages and/or sources along with different domains, the performance of a model also seems to increase. The combination of different domains, languages, and sources (Multi-Cross) seems to further enhance the overall performance of a framework by optimizing the categorization flexibility of the detection model. We also observe that in Multi-Cross approaches, there seems to be a relative rise in the number of data-preprocessing features. Most used features include Word Count, N-Grams, and Punctuation. 

Moreover, through our comparison, we deduce that Cross-Source deception detection appears to be the least effective. This seems to be due to the minimal effect multiple sources (in terms of writing style, text length etc.) have on the model’s performance.

**Figure 4:**
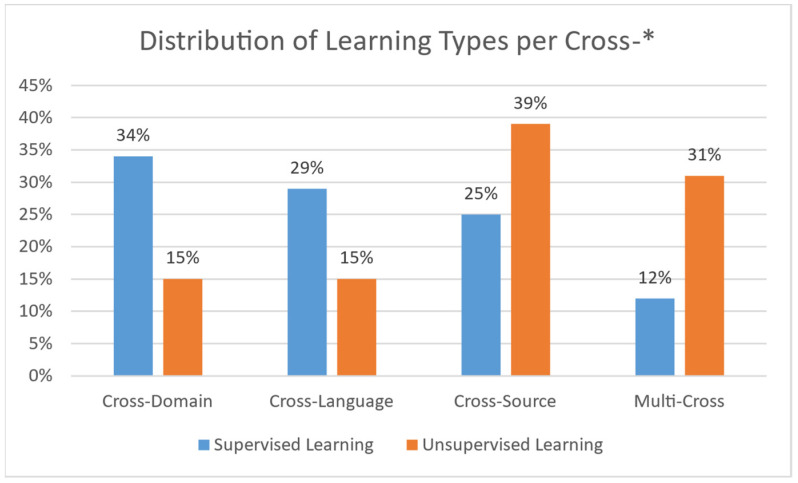
Distribution of learning types per Cross-* category.

**Figure 5:**
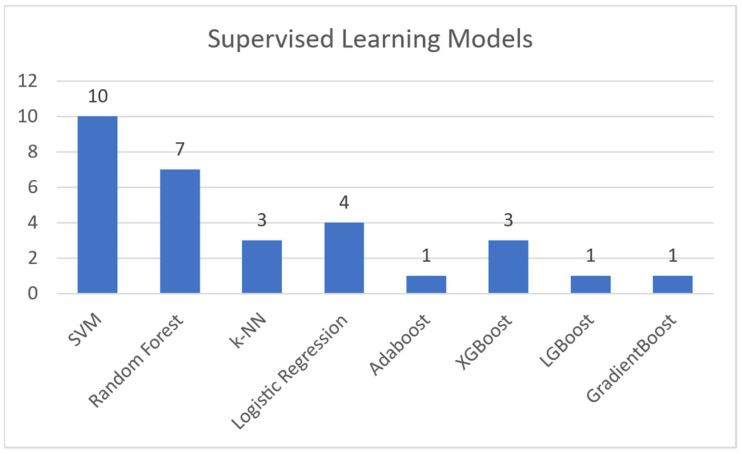
Supervised learning techniques used in misinformation.

**Figure 6:**
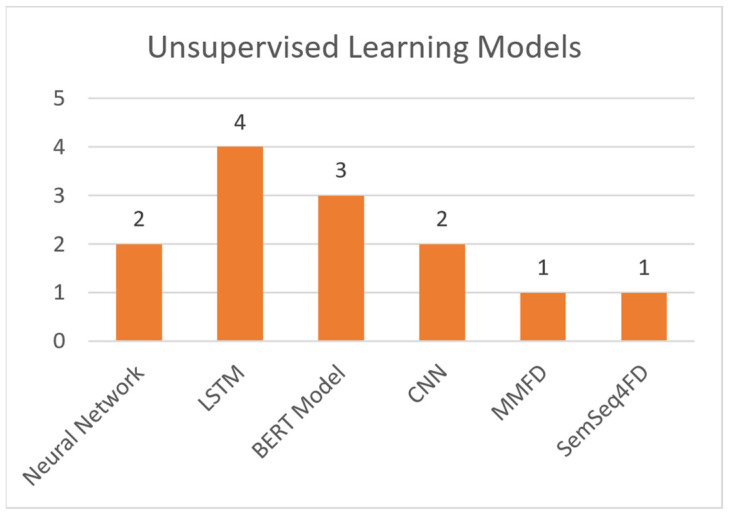
Unsupervised learning techniques used in misinformation.

**Figure 7:**
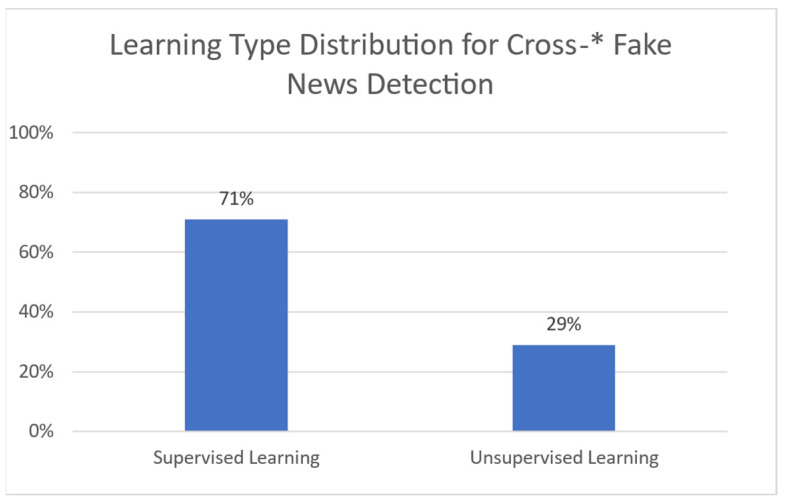
Distribution of learning techniques for Cross-* Detection.

Figure 4, Figure 5 and Figure 6 summarize the distribution of learning techniques (Supervised, Unsupervised Learning) across each cross category, while providing further information regarding the models used for the cross-* detection of misinformation. We notice a preference towards supervised learning in Cross-Domain and Cross-Language detection, whereas Unsupervised Learning prevails in Cross-Source and Multi-Cross. Finally, as depicted in Figure 7, the majority of the methodologies use supervised learning for the deception detection.

**Figure 8:**
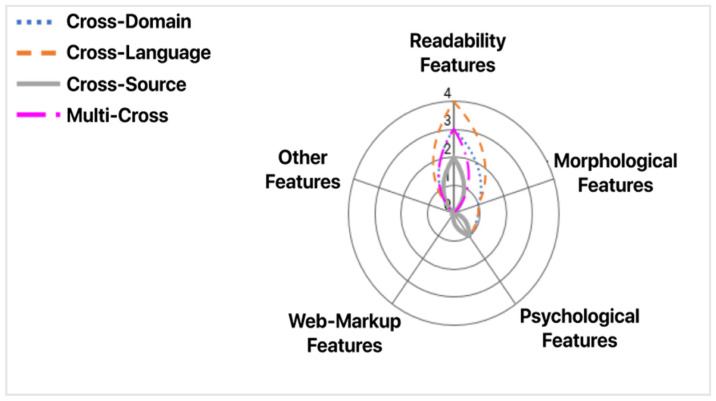
Average number of data pre-processing features used per Cross-Category.

The chart in Figure 8 presents the average number of features per feature category. Specifically, we calculated the average number that all approaches of a cross-category used. On average, all of the cross-categories mostly use readability features during the data pre-processing phase, whereas the web-markup features are the least used. We notice that the Cross-Language approach utilizes the most readability features. This is possibly due to the necessity for creating common ground between languages. A machine learning model will most likely not comprehend testing data in a different language. Breaking information down into its bare components may aid in the recognition of context and semantics within a text of a different language than that of the training set. The Cross-Source approach utilizes the least readability features but does, however, take advantage of psychological features. This seems to be due to the reduced interest in readability and increased focus on emotions that writers, from varying sources, are attempting to induce from their readers.

In the Appendix (see Appendix A) we present a thorough comparison of the cross-* misinformation detection methodologies that we examined in this survey.

## 6. Suggestions, Conclusions and Future Plans

### 6.1. Suggestions and Future Research

We went through a multitude of cross-* methodologies. We conducted an extensive comparison of these approaches and provide helpful insights about the field of deception detection. Each approach possesses a specific set of characteristics which assist or diminish its success rate.

For the comparison, we needed an abundance of consistent and accurate data. Unfortunately, we noticed a major lack of information in several critical points of interest. The most common missing details were the performance metrics, the process of model hyperparameter tuning, the sampling methods, and the data pre-processing features used, as well as the subjects covered in each dataset. The difficulty of retrieving all the necessary information led us to propose a number of good practices—research directions. These practices provide an overview of the information that are crucial in the assessment of a model’s performance. Our aim is also to highlight and explain why this absence of information does not help the research community to form an opinion about the existing approaches.

Trending research indicates that the use of specific data pre-processing features enhances the performance of a method. Such features may be: (i) readability features related to stop word removal and word count, (ii) psychological features regarding to punctuation, as well as (iii) morphological features and especially N-Grams. Additionally, as a learning approach, the majority of these research papers use supervised learning, which seems to be a fairly promising option/choice.

The following five (5) research directions are an underlying basic model for optimizing misinformation detection, based on the best criteria, features, and metrics gathered from all relevant publications.

**Good practice—research direction #1:** Authors should calculate and present all the important metrics for the performance evaluation of a methodology. The most critical metrics are: (i) Accuracy, (ii) F1-Score, (iii) Precision, and (iv) Recall. A great number of published approaches calculates only the metric of Accuracy. This metric alone does not provide enough information to gauge the aptitude of a methodology. It only informs about all the correctly classified cases. In order to gain a thorough view of a model’s performance, more metrics are needed. The second most important metric is the F1-Score, which is the harmonic mean of the Precision and Recall metrics. F1-Score provides more information about the incorrectly classified cases. Possessing both the Accuracy and the F1 metric allows for a clearer view of the effectiveness of a methodology.

**Good practice—research direction #2:** Authors should acknowledge the strengths and weaknesses of an examined model. When we opt for a model, it is crucial to also take into account the requirements it has. There is no specific list of resources that are necessary when constructing a misinformation detection method. Each model is different and thus has different needs. These needs refer to the resources that are necessary, including the size of the dataset that an approach uses, the time needed for the training phase, or even the necessary computing power. Failing to acknowledge and apply these parameters will result in subpar model performance. Therefore, it is advisable for researchers to adjust the resources allocated to their approaches accordingly, in order to achieve better results. For instance, SVM-based techniques are resource heavy. This means that these techniques need a greater number of resources compared to others and, therefore, a larger dataset will more than likely allow for better performance.

**Good practice—research direction #3:** Researchers should retain a balance regarding the amount of data pre-processing features that they use in their methodology. A larger number of features does not necessarily equate to higher all-round performance. This misconception often worsens the evaluation scores of a method, since researchers tend to over-engineer feature sets in an attempt to achieve better results and model flexibility. Researchers should discover and use only the features that fit their approach to boost its performance. Hence, one factor that leads to better results is appropriate feature selection. Another factor is to detect the number of features that are truly necessary for a model. The redundancy of features complicates the whole process and consequently does not improve the detection of fake news.

**Good practice—research direction #4:** Authors should present more details about the hyperparameter tuning that they performed. Hyperparameter tuning refers to the process of selecting a set of optimal hyperparameters for a learning algorithm. A hyperparameter is defined as a parameter that is used to control the learning process. We suggest that such details are to be publicly available in order researchers to be able to clearly view the steps of a methodology. Moreover, this would pave the way for easier open-source testing. Generally, it is quite helpful when a methodology is well documented. Individuals could use this documentation to understand how the methodology works and are encouraged to develop a better and even more accurate model. 

**Good practice—research direction #5:** Researchers should adapt their methodology based on the cross-category they target. Our research indicated that according to the Cross-category that an approach focuses, there are a few tips that can lead to better results. Specifically, in the case of Cross-Domain category it is preferable to use datasets which cover fewer topics but include more events. When an approach belongs to the Cross-Language category, researchers should carefully choose or even create an appropriate model for the task of the detection. Thus, the model plays an important role for the performance. In the case of Cross-Source category, the feature selection was indicated as the most defining factor for higher performance. Finally, in the Multi-Cross methodologies the most important factor is maintaining a balance between datasets, models, and features used.

It is essential to evaluate the performance of a model based on common metrics. A model with an accuracy of 100%, however, is unrealistic. The question that each researcher should ask himself/herself is “At what point do I start blindly trusting my model?”. A global rule for the lowest acceptable value of model’s accuracy does not exist. For example, if a model reached 89% accuracy, this means that there is a 11% possibility for the predictions to be wrong (this statement does not consider the model being lucky in its predictions). For instance, after 1,000,000 predictions, this particular model will have made roughly 110,000 false predictions. Is such percentage of failure acceptable for a method? How can we define the minimum acceptable score? All these questions cannot be answered immediately. The reason that there is not a specific score that determines the perfect accuracy is because it depends on the corresponding methodology and the models that it uses. 

Although, the Accuracy metric depends on the model that is used, it will be quite interesting if researchers could define the minimum acceptable score that will make an approach broadly reliable. Additionally, the acceptable margin of error should be also defined. Using the Accuracy metric as a guideline may seem alluring; however, as shown in the example above, it is not without its fault.

### 6.2. Conclusions

In this paper, we analyze and review the currently prevalent methodologies for detection of misinformation. We especially focus on Cross-Domain, Cross-Language, and Cross-Source methodologies. Moreover, we review methods that take cross-* deception detection a step further, by combining more than one cross-categories, which we call “Multi-cross”. 

In the Appendix (see Appendix A), we present an extensive comparison of all the aforementioned methods. We give details about the sectors that each method is associated with, the techniques that the methods make use of for feature handling, the evaluation metrics used to assess their models, as well as the learning method that they choose. Moreover, we mention whether authors propose a technique for (1) the feature engineering, (2) the feature selection, and (3) the hyper-parameter selection, or they use an existing one. We preliminarily rate the adaptability of all the examined approaches. Specifically, we take into consideration multiple variables such as performance evaluation metrics, data-preprocessing features, datasets used, as well as the domains/languages/sources that each approach includes in the training and testing phase.

The results of our comparison indicate that it is necessary to retain a balance when selecting data pre-processing features. There is a misconception that a greater number of features will definitely lead to higher performance. As pointed out in Section 6 (Good Practice-research direction 3), this is not a true statement, since it is important to choose those features that are necessary for the aim that our model serves. Additionally, the results assist in understanding the factors that may affect the performance of a cross-* approach. In detail, regarding the cross-category we target, there are specific points to which we should pay attention: (1) in Cross-Domain methodologies, the datasets should include enough events; (2) in Cross-Language methodologies, the model is a fairly important component; (3) in Cross-Source methodologies, feature selection is the defining element; and (4) in Multi-Cross methodologies, the crucial factor is the balance among datasets, models, and features that are used.

The points mentioned above led us to propose a set of good practices—research directions—that highlight the type of information that a researcher should mention when a misinformation detection method is presented. Finally, we introduced a few additional cross-* datasets that can be used in cross-* methodologies. 

Overall, our primary aim is to increase the situational awareness regarding misinformation and encourage the research community to further optimize the deception detection by considering more than one dimension of this rising problem.

### 6.3. Future Plans

Future research in this field should consider the different parameters and modifications necessary to optimize model performance and follow a consistent approach to data representation. With this in mind, cross-* misinformation detection remains an evolving field and possesses a community that shows significant promise in creating a successful new method. 

Our future plans include, inter alia, the co-operation with a team of researchers on powerful and promising misinformation detection technologies (e.g., machine learning, etc.), with an eye towards specifying, pilot developing and testing an innovative Cross-Domain methodology that is capable of efficiently fighting misinformation in practice.

## Figures and Tables

**Table 1:** Survey Methodology Attributes.

Goal	Question	Keyword Search
Discover Cross-Domain methodologies for fake news detection	Which of the published methods are addressed to detect fake news in different topics?	(“fake news” OR “disinformation” OR “misinformation” OR “false information”) AND (“cross domain” OR “different domains” OR “different topics” OR “cross domain datasets”) AND (“deception detection” OR “automatic detection” OR “manual detection”)
Discover Cross-Language methodologies for fake news detection	Which of the published methods are addressed to detect fake news in different languages?	(“fake news” OR “disinformation” OR “misinformation” OR “false information”) AND (“cross language” OR “different languages” OR “multilingual”) AND (“deception detection” OR “automatic detection” OR “manual detection”)
Discover Cross-Source methodologies for fake news detection	Which of the published methods are addressed to detect fake news from different sources?	(“fake news” OR “disinformation” OR “misinformation” OR “false information”) AND (“cross source” OR “different sources”) AND (“deception detection” OR “automatic detection” OR “manual detection”)

**Table 2:** Common Evaluation Metrics.

Metric	Definition
Accuracy	Number of correctly predicted articles proportionate to the total amount of texts given to the model for testing.
Recall	Number of correctly predicted Positives (or Negatives) relative to the total amount of correct predictions.
Precision	Number of True Positives (or True Negatives) the model correctly predicted in relation to the total number of Positives (or Negatives) the model predicted.
F1	Describes the balance between Precision and Recall. A model is considered perfect when it achieves an F1 score of 1 and useless if it reaches a value near to 0.

**Table 3:** Categories of Data Pre-processing Features.

Feature Category	Definition
Readability Features	• Information that simplifies/complicates reading comprehension. • Syntax, Tokenization, Average word size, Common Word Count, Sentence Structure, Word Count, Stop Word Removal, Other.
Web-markup Features	• Information regarding the layout of the web pages from which it was gathered.
Morphological Features	• Information related to the grammatical and syntactic structure of sentences. • N-Grams, POS Tags, Misspelled Words, Verb Tense Analysis.
Psychological Features	• Information associated with semantic data that are captured from textual analysis. • Punctuation, Emotional Words, Other.
Other Features	• Features that do not belong in the previous categories. • Style, Plagiarism, Vocabulary.

**Table 4:** Performance Evaluation of Cross-Domain Methodologies.

Ref. No.	Authors	Datasets	Model	Average Accuracy	Precision	Recall	F1-Score
[47]	V. Pérez-Rosas (Univ. of Michigan), B. Kleinberg (Univ. of Amsterdam), A. Lefevre Univ. of Michigan), R. Mihalcea (Univ. of Michigan)	FakeNewsAMT Celebrity	Custom SVM	0.7350	0.7375	0.7350	0.7325
[50]	J. Lin (Louisiana State University), G. Tremblay-Taylor (Keene State College), G. Mou, Di You, K. Lee (Worcester Polytechnic Institute)	PolitiFact GossipCop	Logistic Regression	0.8097	0.8207	0.8097	0.8137
			SVM	0.7677	0.7037	0.6470	0.7973
			KNN	0.8163	0.8133	0.8163	0.7683
			Random Forest	0.8420	0.8443	0.8420	0.8290
			AdaBoost	0.8277	0.8207	0.8277	0.8200
			XGBoost	0.8583	0.8550	0.8583	0.8517
			LSTM-ATT	0.8120	0.8167	0.8120	0.8123
[49]	G. Jerripothula K. (Indraprastha IT Institute)	FakeNewsAMT Celebrity	Spinbot + Grammarly + GloVe	0.6300	N/A	N/A	N/A
[35]	T. Saikh, A. Ekbal, P. Bhattacharayya (Indian Institute of Technology), A. De (Government College of Engineering, Berhampore)	FakeNewsAMT Celebrity	MLP ELMo	0.7680 0.8115	N/A	N/A	N/A
[33]	S. Castelo, A. Santos, K. Pham, J. Freire, A. Elgahafari (NYU), T. Almeida, E. Nakamura (Federal Univ. of Amazonas)	US Election 2016 Celebrity	FNDetector (SVM) FNDetector (KNN) FNDetector (RF) TAG Model (SVM) TAG Model (KNN) TAG Model (RF)	0.5900 0.5750 0.5350 0.6650 0.6200 0.6200	N/A	N/A	N/A
[51]	Sebastian Kula, Michal Choras, Rafal Kozik, Pawel Ksieniewicz1, Michal Wo’zniak (Univ. of Science and Technology, Kazimierz Wielki Univ., Wroclaw Univ. of Science and Technology, Poland)	ISOT Kaggle	RNN	0.9986	0.9982	0.9991	0.9986
[52]	Kai Shu, Deepak Mahudeswaran, Suhang Wang, Huan Liu (Computer Science and Engineering, Arizona State Univ., Penn State Univ.)	FakeNewsNet PolitiFact GossipCop	Multiple Different Models (RF, Naïve Bayes, Decision Trees, Logistic Regression)	0.8520	0.8440	0.8660	0.8520

**Table 5:** Data Pre-Processing Features used in Cross-Domain Methodologies.

Features		Pérez-Rosas et al.	Gautam A. et al.	Saikh T. et al.	Castelo S. et al.	Lin J. et al.
Readability Features	Syntax	✓	✓			
	Tokenization					
	Average Word Size	✓				
	Common Word Count	✓	✓			
	Sentence Structure	✓	✓			✓
	Word Count	✓	✓	✓	✓	✓
	Stop Word Removal					
	Other readability features	✓	✓		✓	
Psychological Features	Punctuation	✓	✓			✓
	Emotional Words	✓				
	Other Psychological features	✓			✓	✓
Morphological Features	N-Grams	✓		✓	✓	✓
	POS Tags	✓				
	Misspelled Words		✓			
	Verb Tense Analysis		✓			
Others	Style		✓			
	Plagiarism		✓			
	Vocabulary		✓			
Web Markup Words					✓	

**Table 6:** Domains Covered by Cross-Domain Methodologies.

Suggested by	Education	Entertainment	Business	Politics	Technology	Sports	Celebrity	Other
Pérez-Rosas et al.	✓	✓	✓	✓	✓	✓	✓	
Gautam A. et al.	✓	✓	✓	✓	✓	✓	✓	
Saikh T. et al.	✓	✓	✓	✓	✓	✓	✓	
Castelo S. et al.				✓			✓	
Lin J. et al.				✓			✓	
Kula et. al.				✓				World News
Shu et al.				✓			✓	

**Table 7:** Adaptability of Cross-Domain Methodologies.

Suggested by	1st Parameter (Accuracy)	2nd Parameter (# of Sectors)	3rd Parameter (# of Features)	4th Parameter (# of Datasets)	Overall Adaptability
Pérez-Rosas et al.	Relatively flexible	Flexible	Relatively flexible	Relatively flexible	Relatively flexible
Gautam A. et al.	Relatively flexible	Flexible	Relatively flexible	Relatively flexible	Relatively flexible
Saikh T. et al.	Flexible	Flexible	Non-flexible	Relatively flexible	Flexible
Castelo S. et al.	Relatively flexible	Non-flexible	Non-flexible	Non-flexible	Non-flexible
Lin J. et al.	Flexible	Non-flexible	Non-flexible	Non-flexible	Non-flexible

**Table 8:** Performance Evaluation of Cross-Language Methodologies.

Ref. No.	Authors	Datasets	Model	Average Accuracy	Precision	Recall	F1-Score
[37]	S. W. Chu, R. Xie, Y. Wang (Univ. of Hong Kong)	English (Custom) Chinese (Custom)	BERT Model	0.6701	0.6750	0.8700	0.7400
[53]	I. Vogel, M. Meghana (Fraunhofer Institute)	Custom Dataset	CNN	0.7250	0.7850	0.6600	0.7150
			SVM	0.7500	0.8200	0.6450	0.7200
			Logistic Regression	0.7400	0.7950	0.6400	0.7100
[54]	H. Queiroz Abonizio, J. de Morais, S. Barbon (State Univ of Londrina), G. Marques Tavares (Univ. of Milan)	FNC	Random Forest	0.8530	N/A	N/A	N/A
[55]	G. Guibon(Aix-Marseille Univ.), L. Ermakova (Univ. de Bretagne), H. Seffih (GeolSemantics), A. Firsov (Knoema-Corp.), G. Le Noé-Bienvenu (PluriTAL)	Custom Dataset	Decision Tree SVM Custom LGBM Optimized LGBM Optimized SVM CNN	N/A	N/A	N/A	0.5776 N/A 0.8476 0.8727 0.8835 N/A

**Table 9:** Data Pre-Processing Features Used in Cross-Language Methodologies.

Features		Chu et al.	Vogel et al.	Abonizio et al.	Guibon et al.
Readability Features	Syntax		✓	✓	✓
	Tokenization			✓	✓
	Average Word Size		✓	✓	
	Common Word Count	✓		✓	✓
	Sentence Structure			✓	
	Word Count		✓	✓	✓
	Stop Word Removal		✓		✓
	Other readability features				
Psychological Features	Punctuation			✓	✓
	Emotional Words		✓		
	Other Psychological features		✓	✓	
Morphological Features	N-Grams				✓
	POS Tags			✓	
	Misspelled Words				
	Verb Tense Analysis		✓	✓	
Others	Style		✓	✓	
	Plagiarism				
	Vocabulary				
Web Markup Words					✓

**Table 10:** Languages Per Cross-Language Methodology.

Suggested by	Languages
Chu S. et al.	English, Chinese
Vogel et al.	English, Spanish
Abonizio et al.	English, Spanish, Portuguese
Guibon et al.	English, French

**Table 11:** Adaptability of Cross-Language Methodologies.

Suggested by	1st Parameter (Accuracy)	2nd Parameter (Num. of Sectors)	3rd Parameter (Num. of Features)	4th Parameter (Num. of Datasets)	Overall Adaptability
Chu S. et al.	Relatively flexible	Relatively flexible	Non-flexible	Non-flexible	Non-flexible
Vogel et al.	Relatively flexible	Relatively flexible	Relatively flexible	Non-flexible	Relatively flexible
Abonizio et al.	Flexible	Flexible	Relatively flexible	Relatively flexible	Flexible
Guibon et al.	Non-flexible	Relatively flexible	Non-flexible	Non-flexible	Non-flexible

**Table 12:** Performance Evaluation of Cross-Source Methodologies.

Ref. No.	Authors	Datasets	Model	Average Accuracy	Precision	Recall	F1-Score
[59]	L. Wang, X. Shen, G. Weikum (Max Planck Institute for Informatics), L. Wang (Shandong University), G. de Melo (Hasso Plattner Institute (University of Potsdam)	Custom Dataset	LR	N/A	0.5300	0.3412	0.3540
			SVM		0.5132	0.3617	0.3840
			LSTM		0.4715	0.5772	0.4745
			LSTMR		0.4797	0.3317	0.3767
[57]	Y.-H. Huang, F. Calderon, T.-W. Liu, Y.-S. Chen, S.-R. Lee (National Tsing Hua University)	FRN KJR NELA-GT 2018	GBT	0.6683	N/A	N/A	N/A
			SVM	0.6860			
			Random Forest	0.6026			
			Bi-LSTM-attention	0.6658			
[58]	H. Karimi, P. C. Roy, S. Saba-Sadiya, J. Tang (Michigan State University)	LIAR	Basic SVM Basic Random Forest Basic NN MMFD	0.2998 0.2701 0.2912 0.3881	N/A	N/A	N/A
[56]	F. T. Asr, M. Taboada (Simon Fraser University)	Custom Dataset	SVM	N/A	N/A	N/A	0.8550

**Table 13:** Data Pre-Processing Features Used by Cross-Source Methodologies.

Features		Wang et al.	Huang et al.	Karimi Et Al.	Asr et al.
Readability Features	Syntax		✓		
	Tokenization		✓		
	Average Word Size				✓
	Common Word Count				✓
	Sentence Structure		✓		✓
	Word Count				✓
	Stop Word Removal				✓
	Other readability features				
Psychological Features	Punctuation				✓
	Emotional Words				✓
	Other Psychological features				✓
Morphological Features	N-Grams		✓	✓	✓
	POS Tags		✓		✓
	Misspelled Words				
	Verb Tense Analysis				
Others	Style		✓		
	Plagiarism				
	Vocabulary				
Web Markup Words		✓			

**Table 14:** Adaptability of Cross-Source Methodologies.

Suggested by	1st Parameter (Accuracy)	2nd Parameter (Num. of Sectors)	3rd Parameter (Num. of Features)	4th Parameter (Num. of Datasets)	Overall Adaptability
Wang et al.	Non-flexible	Relatively flexible	Relatively flexible	Relatively flexible	Relatively flexible
Huang et al.	Relatively flexible	Flexible	Relatively flexible	Relatively flexible	Relatively flexible
Karimi et al.	Non-flexible	Relatively flexible	Non-flexible	Relatively flexible	Non-flexible
Asr et al.	Non-flexible	Relatively flexible	Non-flexible	Relatively flexible	Non-flexible

**Table 15:** Performance Evaluation of Multi-Cross Methodologies.

Ref. No.	Authors	Datasets	Model	Average Accuracy	Precision	Recall	F1-Score
[6]	Yuhang Wang, Li Wang, Yanjie Yang, Tao Lian (Taiyuan University of Technology)	SLN (English) LUN (English) Weibo (Chinese) RCED (Chinese)	SemSeq4FD	0.8825	0.8900	0.8800	0.8967
[48]	Gautam Kishore Shahi (Univ. of Duisburg-Essen), Durgesh Nandini (Univ. of Bamberg)	FakeCovid	BERT-based Classification	N/A	0.7800	0.7500	0.7600
[46]	Caio Libanio Melo Jeronimo, Leandro Balby Marinho, Claudio Campelo, Adriano Veloso, Allan Sales da Costa Melo (Federal Univ. of Campina Grande)	Custom Dataset	XGBoost Random Forest Dummy	0.8350 0.8950 0.8000	N/A	N/A	N/A

**Table 16:** Data Pre-Processing Features Used by Multi-Cross Methodologies.

Features		Y. Wang et al.	Jeronimo et al.	Shahi G. & Nandini D.
Readability Features	Syntax			
	Tokenization	✓		✓
	Average Word Size	✓		✓
	Common Word Count	✓		
	Sentence Structure		✓	
	Word Count	✓	✓	✓
	Stop Word Removal	✓	✓	
	Other readability features			
Psychological Features	Punctuation		✓	
	Emotional Words		✓	
	Other Psychological features			
Morphological Features	N-Grams	✓		
	POS Tags			
	Misspelled Words			✓
	Verb Tense Analysis			
Others	Style			
	Plagiarism			
	Vocabulary			
Web Markup Words				✓

**Table 17:** Domains Covered by Multi-Cross Methodologies.

Suggested by	Education	Entertainment	Business	Politics	Technology	Sports	Celebrity	Other
Y. Wang et al.	✓	✓		✓	✓			Military, Health, Economy, Society
Jeronimo C. L. M. et al.				✓		✓		Economy, Culture
Shahi, G. & Nandini, D.								Health, Science

**Table 18:** Cross-Categories of Multi-Cross Methodologies.

Suggested by	Cross-Domain	Cross-Language	Cross-Source
Y. Wang et al.	✓	✓	✓
Jeronimo C. et al.	✓		✓
Shahi, G., & Nandini, D.	✓	✓	✓

**Table 19:** Adaptability of Multi-Cross Methodologies.

Suggested by	1st Parameter (Accuracy)	2nd Parameter (Num. of Sectors)	3rd Parameter (Num. of Features)	4th Parameter (Num. of Datasets)	5th Parameter (Num. of Categories)	Overall Adaptability
Y. Wang at al.	Flexible	Flexible	Relatively flexible	Relatively flexible	Flexible	Flexible
Jeronimo C. et al.	Non-flexible	Relatively flexible	Non-flexible	Relatively flexible	Relatively flexible	Relatively flexible
Shahi, G., & Nandini, D.	Flexible	Non-flexible	Relatively flexible	Relatively flexible	Flexible	Flexible

## Data Availability

Publicly available datasets were analyzed in this study. This data can be accessed via links that are referred to in the reference section.

## Appendix A

**Table A1:** A Thorough Comparison of Cross-* Misinformation Detection Methodologies.

Authors	Publication Title	Cross-*	Sectors (Domain/Language/Source)	Dataset	Data Pre-Processing	Word Vector Representation	Machine Learning	Performance Metric
PÉREZ-ROSAS et al., Univ. of Michigan, Univ. of Amsterdam	Automatic detection of fake news	Cross-Domain	Business, Education, Politics, Sports, Technology, Entertainment	FakeNewsAMT Celebrity	Syntax, N-Grams, Punctuation, Psychological, Readability, Word Count, Sentence Structure, Emotional Words, Common Word Count, Average Word Size, POS Tags	Bag of Word	supervised learning	A (Accuracy), F1, R (Recall), P (Precision)
GAUTAM A. and JERRIPOTHULA., Indraprastha Institute of Information Technology Delhi	SGG: Spinbot, grammarly and GloVe based fake news detection	Cross-Domain	Business, Education, Politics, Sports, Technology, Entertainment	FakeNewsAMT Celebrity	Syntax, Punctuation, Readability, Word Count, Misspelled Words, Verb Tense Analysis, Sentence Structure, Style, Plagiarism, Vocabulary, Common Word Count	TF-IDF vectorizer	supervised learning	A (Accuracy)
SAIKH T. et al. Indian Institute of Technology Patna, Government College of Engineering	A deep learning approach for automatic detection of fake news	Cross-Domain	Business, Education, Politics, Sports, Technology, Entertainment	FakeNewsAMT Celebrity	N-Grams, Word Count	N/A	supervised learning	A (Accuracy)
CASTELO S. et al., New York Univ., Federal Univ. of Amazonas	A topic-agnostic approach for identifying fake news pages	Cross-Domain	Political News, Entertainment	US Election 2016 Celebrity	N-Grams, Psychological, Readability, Word Count, Web Markup Songs	Bag of Words and TF-IDF	supervised learning	A (Accuracy)
LIN J. et al. Louisiana State Univ., Keene State College, Worcester Polytechnic Institute	Detecting fake news articles	Cross-Domain	Political News, Entertainment	PolitiFact GossipCop	N-Grams, Punctuation, Psychological, Readability, Word Count, Sentence Structure	Bag of Words and TF-IDF	deep learning	A (Accuracy), F1, R (Recall), P (Precision)
CHU S. et al. Univ. of Hong Kong	Cross-language fake news detection	Cross-Language	English, Chinese	English (Custom) Chinese (Custom)	Common Word Count	N/A	supervised learning	A (Accuracy), F1, R (Recall), P (Precision)
VOGEL et al. Fraunhofer Institute SIT	Detecting fake news spreaders on Twitter (multilingual perspective)	Cross-Language	English, Spanish	Custom Dataset	Syntax, Psychological, Word Count, Verb Tense Analysis, Style, Stop Word Removal, Emotional Words, Average Word Size	Bag of Words and TF-IDF	supervised learning	A (Accuracy), F1, R (Recall) P (Precision)
ABONIZIO et al. State Univ. of Londrina, Univ. degli Studi di Milano	Language-independent fake news detection: English, Portuguese and Spanish mutual features	Cross-Language	English, Spanish, Portuguese	FNC	Syntax, Punctuation, Psychological, Word Count, Verb Tense Analysis, Sentence Structure, Style, Common Word Count, Average Word Size, Tokenization, POS tags	Bag of Word	supervised learning	A (Accuracy)
GUIBON et al. Aix-Marseille Univ., Univ. de Bretagne Occidentale, Geol Semantics, Knoema-Corporation Perm, PluriTAL	Multilingual fake news detection with satire	Cross-Language	English, French	Custom Dataset	Syntax, N-Grams, Punctuation, Word Count, Stop Word Removal, Web Markup Words, Common Word Count, Tokenization	TF-IDF vectorizer	supervised and deep learning	F1
WANG L. et al. Max Planck Institute for Informatics, Shandong University, Univ. of Potsdam	Cross-Domain learning for clarifying propaganda in online contents	Cross-Source	Speeches, News and Tweets from various sources	Custom Dataset	Web Markup Words	Bag of Words and TF-IDF	supervised and deep learning	P (Precision) R (Recall) F1
ASR et al. Simon Fraser University	The data challenge in misinformation detection: Source reputation vs. content veracity	Cross-Source	1st (The Natural News, Activist Report, The Onion, The Borowitz Report, Clickhole, America News, DC Gazette, Gigaword News), 2nd (The Onion, The Beaverton, The Toronto Star, The New York Times), 3rd (Multiple FB pages), 4th (multiple sources, by Snopes)	Rashkin, Rubin, BuzzFeedUSE, Snopes312	N-Grams, Punctuation, Psychological, Word Count, Sentence Structure, Stop Word Removal, Emotional Words, Common Word Count, Average Word Size, POS Tags	Bag of Words and TF-IDF	supervised learning	F1
HUANG et al. National Tsing Hua University	Conquering cross-source failure for news credibility: Learning generalizable representations beyond content embedding	Cross-Source	1st dataset FRN (NPR, New York Times, WallStreet Journal, Bloomberg), 2nd dataset KJR (Before It’s News, Daily Buzz Live, Activist Post, BBC, Reuters, CNN, ABC News, NYTimes) 3rd dataset NELA (195 sources)	FRN KJR NELA	Syntax, N-Grams, Sentence Structure, Style, Tokenization, POS Tags	TF-IDF vectorizer	supervised and deep learning	A (Accuracy)
KARIMI et al. Michigan State University	Multi-source multi-class fake news detection	Cross-Source	LIAR LIAR (From multiple sources selected by Politifact)	LIAR	N-Grams	Bag of Word	supervised and deep learning	A (Accuracy)
SHU et al., Arizona State Univ., Penn State University	Hierarchical propagation networks for fake news detection: Investigation & exploitation	Cross-Domain	Domains (Political News, Entertainment)	FakeNewsNet, PolitiFact, GossipCop	Custom	N/A	supervised learning	A(Accuracy), P(Precision), R(Recall)
Y. WANG et al. Taiyuan Univ. of Technology	SemSeq4FD: Integrating global semantic relationship & local sequential order to enhance text representation for fake news detection	Multi-Cross	Domains (health, economic, technology, entertainment, society, military, political and education), Languages (English, Chinese), Sources (The Onion, Gigaword News, Associated Press, Washington Post, Bloomberg NewsWire, The Borowitz Report, Clickhole, Toronto Star, NY Times, The Beaverton)	SLN (English) LUN (English) Weibo (Chinese) RCED (Chinese)	N-Grams, Word Count, Stop Word Removal, Common Word Count, Average Word Size, Tokenization	TF-IDF vectorizer	supervised and deep learning	A (Accuracy), F1, R (Recall) P (Precision)
JERONIMO C. et al. Federal Univ. of Campina Grande	Fake news classification based on subjective language	Multi-Cross	Domains (Politics, Sports, Economy, Culture) Languages (English, Portuguese), Sources (Fohla de Sao Paolo, Estadao)	Custom Dataset	Punctuation, Word Count, Sentence Structure, Stop Word Removal, Emotional Words	TF-IDF vectorizer	supervised learning	A (Accuracy)
SHAHI, G. K., and NANDINI, D. Univ. of Duisburg-Essen, Univ. of Bamberg	FakeCovid—A multilingual cross domain FactCheck news dataset for COVID-19	Multi-Cross	Domains (Health, Science) Languages (English, Hindi, German), Source (Multiple sources from Snopes and Poynter)	FakeCovid	Word Count, Mispelled Words, Web Markup Words, Average Word Size, Tokenization	N/A	N/A	P (Precision), R (Recall) F1
KULA et al., UTP Univ. of Science and Technology, Kaszimierz Wielki Univ., Wroclaw	Sentiment analysis for fake news: Detection by means of neural networks	Cross-Domain	Domains (World News, Politics, US news)	ISOT, Kaggle	Custom	N/A	proposed	A(Accuracy), P(Precision), R(Recall), F1-Score
